# Carbonate precipitation and phosphate trapping by microbialite isolates from an alkaline insular lake (Bagno dell'Acqua, Pantelleria Island, Italy)

**DOI:** 10.3389/fmicb.2024.1391968

**Published:** 2024-05-22

**Authors:** Cristina Mazzoni, Agnese Piacentini, Letizia Di Bella, Luca Aldega, Cristina Perinelli, Aida Maria Conte, Michela Ingrassia, Tania Ruspandini, Andrea Bonfanti, Benedetta Caraba, Francesco Giuseppe Falese, Francesco Latino Chiocci, Stefano Fazi

**Affiliations:** ^1^Department of Biology and Biotechnology “C. Darwin”, Sapienza University of Rome, Rome, Italy; ^2^Water Research Institute, National Research Council (IRSA-CNR), Montelibretti, Rome, Italy; ^3^Department of Earth Sciences, Sapienza University of Rome, Rome, Italy; ^4^Institute of Environmental Geology and Geoengineering, National Research Council (IGAG-CNR), Department of Earth Sciences, Sapienza University of Rome, Rome, Italy; ^5^National Biodiversity Future Center (NBFC), Palermo, Italy

**Keywords:** biomineralization, alkaline lake, microbialites, pantelleria, phosphate trapping, hazenite

## Abstract

The Bagno dell'Acqua lake is characterized by CO_2_ emissions, alkaline waters (pH = 9) and Eh values which indicate strongly oxidizing conditions. A typical feature of the lake is the presence of actively growing microbialites rich in calcium carbonates and silica precipitates. Mineralogy, petrography and morphology analyses of the microbialites were coupled with the analysis of the microbial community, combining molecular and cultivation approaches. The DNA sequencing revealed distinct patterns of microbial diversity, showing pronounced differences between emerged and submerged microbialite, with the upper layer of emerged samples exhibiting the most distinctive composition, both in terms of prokaryotes and eukaryotes. In particular, the most representative phyla in the microbial community were Proteobacteria, Actinobacteriota, and Bacteroidota, while Cyanobacteria were present only with an average of 5%, with the highest concentration in the submerged intermediate layer (12%). The role of microorganisms in carbonate mineral formation was clearly demonstrated as most of the isolates were able to precipitate calcium carbonate and five of them were characterized at molecular level. Interestingly, when microbial isolates were cultivated only in filtered water, the precipitation of hazenite was observed (up to 85%), opening new prospective in P (phosphate) recovery from P depleted environments.

## 1 Introduction

The world is facing an unprecedented climate crisis, and carbon emissions are a major contributor to temperature increase. Identifying new carbon sinks is a major asset for climate change mitigation (IPCC, 2022) and new carbon capture technologies are required (Bajpai et al., 2022). In this context, microorganisms, thanks to enzymatic activities such as urease, carbonic anhydrase and Rubisco, can mediate the Microbial Induced Carbonate Precipitation (MICP) (Han et al., 2013), contributing to carbon dioxide sequestration (Mitchell et al., 2010; Okyay et al., 2016). Recently, research activities focused on harnessing the power of microbes for Next-Gen Carbon Capture by the exploration of extreme CO_2_-rich environments. This aims to isolate and characterize microorganisms that have evolved to be exceptionally effective in capturing carbon dioxide (Chen et al., 2021). The ability to rapidly form calcite could also be used for biotechnological applications such as the removal of heavy metals, metalloids, and cations like Ca^2+^ from contaminated soil and water.

An opportunity to deepen the understanding of carbon capture and the role of those microorganisms in the biomineralization process lies in the exploration of microbialites, which are a type of organo-sedimentary rock formed when benthic microbial communities promote the precipitation of authigenic minerals or capture and bind detrital sediments (Moore and Burne, 1987). Through microbially-mediated mineralization and recycling of metabolites, bacteria are able to develop their own geological and biological substrates, which enables these systems to adapt to a wide range of environmental circumstances that have existed throughout Earth's history (Dupraz et al., 2009). Microbialites constitute, therefore, a hot spot for CO_2_ biomineralization, serving as benthic sedimentary rocks composed of carbonate mud (particle diameter < 5 μm).

Microbialites may be associated with specific geological settings, such as ancient reef or geothermal regions. Moreover, these organo-sedimentary rocks are commonly located in shallow aquatic environments such as lakes, lagoons, and coastal areas. Among other environments, modern microbialites can be found in hypersaline lakes (Pace et al., 2016; Bischoff et al., 2020), in crater lakes (Zeyen et al., 2015, 2021), and in alkaline Lakes (Souza-Egipsy et al., 2005), including the Bagno dell'Acqua lake, Pantelleria, Italy (Cangemi et al., 2016). In particular, this latter site exhibits extreme chemical-physical conditions, to the extent that it has been proposed as a Martian analog (Baliva et al., 1999).

Environmental stability, encompassing factors like temperature and water chemistry, proves crucial for the prolonged preservation of microbialites (Warden et al., 2019). Abrupt changes in physico-chemical conditions can disrupt the delicate equilibrium necessary for microbialite growth. Additionally, prevalent characteristics of sites hosting microbialites include elevated salinity and alkaline conditions, the latter of which facilitates carbonate precipitation (Reid et al., 2024).

The formation of microbialites is not completely understood, but a recent study, comparing two sites in Western Australia and the Bahamas, showed that microbialites are not created equal, being the initial architecture determined by a dynamic balance between extrinsic and intrinsic factors (Reid et al., 2024). Despite the focus on biomineralization was primarily on the study of cyanobacteria, some studies provided evidence of the involvement of heterotrophic bacteria in this process (Doemel and Brock, 1974). It has become apparent that microbial communities in microbialites showcase a wide diversity, with members belonging to various taxonomic orders that can exhibit similar metabolism, supporting an “alkalinity machine” (Saghaï et al., 2015). Thus, despite the long-held belief that stromatolites primarily form due to the photosynthetic activity of cyanobacteria, the discovery of anoxygenic photosynthesis in Chloroflexi, also found in calcifying mats and stromatolites, has revealed the significance of other non-phototrophic organisms in these systems (Saghaï et al., 2015; Cangemi et al., 2016). Carbonate precipitation is primarily caused by microbial metabolic activity by three main mechanisms leading to granule formation: (i) biologically influenced mineralization, where mineral precipitation is favored by nucleation on bacterially produced organic polymers (EPS). In this case, the process occurs in a saturated solution and does not require the cells to be metabolically active; (ii) biologically controlled mineralization, where precipitation occurs intracellularly and can occur under seemingly out-of-equilibrium conditions; (iii) biologically induced mineralization, a process in which certain bacteria, such as the ureolytic bacteria, contribute to the formation of carbonate crystals through various metabolic pathways (Görgen et al., 2021). In the complex structure of surface microbialites, diatoms and cyanobacteria can produce cavities (crypts) that contribute to the microenvironment architecture. The growth of a microbialite, incorporating geochemically and biologically induced or influenced precipitations, requires a continuous influx of water to supply ions for mineral growth (Webb and Kamber, 2011). The surface crypts allow the formation of micro-niches conducive to the settlement of bacteria; it is within these microsites that various metabolic processes mediate or influence mineral precipitations (Riding, 2000).

In addition to carbonate precipitation, bacterial activity may be involved in the biomineralization of other minerals, such as those involving phosphorus (Sun et al., 2020). To date, co-precipitation of dissolved phosphorus with calcite has been observed in numerous shallow lakes worldwide (Danen-Louwerse et al., 1995). Given that phosphorus is present in trace amounts in water, while carbonate is a major component, there is competition between phosphorus and carbonate for calcium ions. Consequently, CaCO_3_-CaPO_4_ co-precipitation requires appropriate conditions of temperature, pH, and calcium-to-phosphorus ratio. Yang et al. (2011) investigated the formation of a biogenic phosphate mineral, hazenite, discovered on completely dried or decomposed green algae (cyanobacteria) on porous calcium carbonate substrates (mainly calcite and aragonite) or tufa, along the south shore of Mono Lake (California, USA). Hazenite is a phosphate of potassium, sodium, and magnesium and it was believed to form under unique environmental conditions characterized by high alkalinity, hypersalinity, and elevated pH, with significant involvement of cyanobacteria. Recent studies define hazenite as the first struvite-type compound containing two structurally distinct monovalent cations (K and Na), indicating an exclusive role of the biological activity in the mineralization process (Yang et al., 2011). Hazenite has been observed for the second time at the Belmaco Cave site (La Palma, Spain) (Fernández-Palacios et al., 2023). To date, these two sites appear to be the only ones where this biomineral has been identified. To the best of our knowledge, no evidence of PO_4_ precipitation has been reported in deposits of carbonate mud such as microbialites in oligotrophic environments, and no isolates were described to promote hazenite precipitation.

In the present study, in a CO_2_ enriched alkaline environment, molecular and cultivation techniques were employed for the following objectives:

1) Analyse the microbial diversity in different layers of modern microbialites. In particular, due to the increasing occurrence of water scarcity in the Mediterranean and the consequent changes in water level (Bayoumy et al., 2019), the objective was to compare different layers of the microbialites under different water availability (submerged *vs* emerged);
2) Isolate microbial strains in order to verify the active role of non-phototrophic microorganisms in carbonate precipitation in order to explore MICP could contribute to the overall diagenesis of modern microbialites;
3) Investigate the capability of these microorganisms to co-precipitate phosphates in order to verify the capacity of P recovery from P depleted environments.

## 2 Materials and methods

### 2.1 Sampling

**Figure 1:**
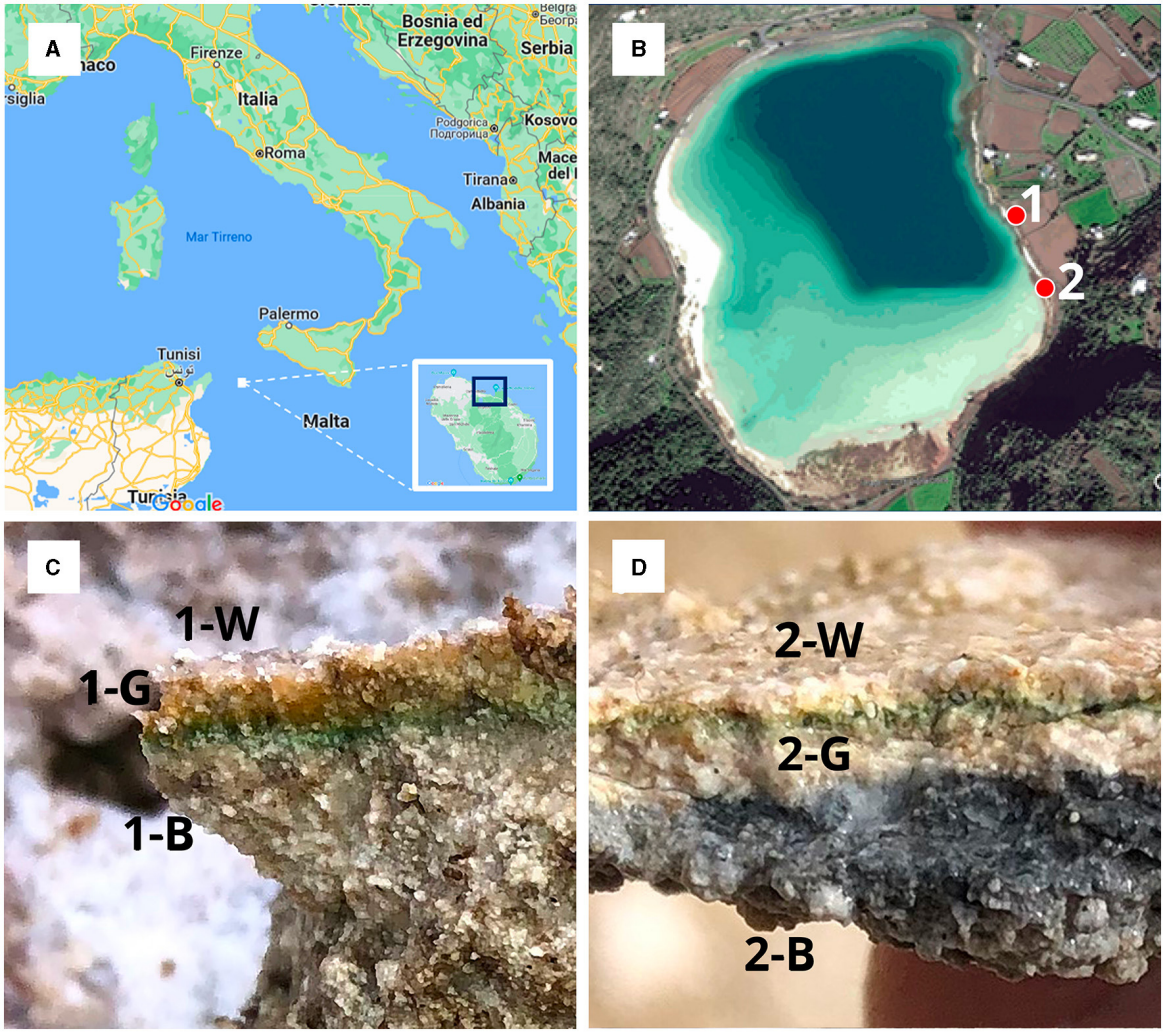
**(A)** Map of Italy with focus on the island of Pantelleria; **(B)** Satellite image reporting sampling locations labeled as 1 (emerged microbialite) and 2 (submerged microbialite); **(C)** emerged microbialite sample with the three analyzed layers (1-W, 1-G, 1-B); **(D)** submerged microbialite sample with the three analyzed layers (2-W, 2-G, 2-B).

The alkaline lake Bagno dell'Acqua (Figures 1A, B) (Pantelleria Island, Italy) is characterized by the presence of microbialites along the entire shore. We collected emerged and submerged partly consolidated microbialites samples (Figures 1C, D) from the eastern shore of the lake (site 1 and 2 emerged and submerged, respectively) into sterile tubes and maintained at 4°C until they were brought to the laboratory. Additional fragments microbialites were immediately frozen (−20°C). For the CARD-FISH analysis other fragments were fixed in ethanol (50% final concentration) (Fazi et al., 2007; Ramm et al., 2012) immediately after collection and keep frozen (−20°C) until further processing. All the microbialites fragments were collected by sterile disposable tweezers by operators wearing gloves. All the samples appear roughly laminated and poorly lithified varying in color. In particular, for each sample three differently colored layers, hereafter named: white upper (W), green middle (G), black lower (B), were observed and selected to be analyzed for texture, mineral assemblage and chemistry.

### 2.2 SEM-EDS and XRD

SEM-EDS and X-ray diffraction (XRD) analyses were used to determine mineral and chemical components of bacteria precipitates and microbialite layers. A descriptive (qualitative and quantitative) approach was used to examine the microbialites by making observations through Scanning Electron Microscope SEM -EDS, FEI Quanta 400, at the SEM Laboratory of the Earth Science Department, Sapienza University of Rome (Italy), in order to obtain their morphological, compositional characteristics, and biological evidences.

The microbialite samples were collected and immediately stored in specific jar in order to avoid any contamination. In the sedimentological laboratory of Earth Sciences Department of Sapienza University of Rome, the sediment fragments were dried in ventilated oven and finally attached to 12.5 mm SEM stubs using carbon tabs. The samples were then gold-coated using a Emitech K550X sputter coater, with a routine cycle time for coating SEM samples with conductive coating (5–15nm) of gold (Au) typically < 4 min. SEM micrographs and EDS analysis were carried out using a standardized method, with an accelerating voltage ranging between 15 and 30 kV in high vacuum mode, hence the focus was adjusted to match the change in working distance (~12 mm) over the same range of the specimen and an improved image was obtained, ranging between 10 and 200 μm resolution. SEM-EDS were employed to obtain chemical analysis and microstructural information, as well as a careful characterization of inclusions and biological evidences.

1. The mineralogical assemblage of bacterial precipitates and 3 microbialite layers was determined by XRD analysis using a Bruker D8 Advance X-ray system equipped with Lynxeye XE-T silicon-strip detector at the Department of Earth Sciences, Sapienza University of Rome (Italy). The X-ray system was operated at 40 kV and 30 mA using CuKα radiation (λ = 1.5406A°). The samples were X-rayed from 2 to 70°2θ, with a step size of 0.02° 2θ while spinning the sample. Bacteria precipitates were hosted in Si-wafers to have a high signal-to-noise ratio. Data were collected with variable slit mode to keep the irradiated area on the sample surface constant and converted to fixed slit mode for semiquantitative analysis. Semiquantitative estimation of minerals was performed by calculating peak areas and using mineral intensity factors as calibration constants (Moore and Reynolds, 1997).

### 2.3 Cross kingdom 16S/18S rRNA gene variable regions 48 (abeV48A) sequencing

Microbialite fragments was crushed by sterile spatula. DNA was extracted from 1 g of crushed microbialite fragments by PowerSoil Isolation Kit (MoBio, Carlsbad, CA) according to the manufacturer's instructions. Amplicon libraries for the archaea/bacteria/eukaryota 16S/18S rRNA gene variable regions 48 (abeV48A) were prepared using a custom protocol. Up to 25 ng of extracted DNA was used as template for PCR amplification, and each PCR reaction (50 μL) contained 0.5 mM dNTP mix, 0.01 units of Platinum SuperFi DNA Polymerase (Thermo Fisher Scientific, USA), and 500 nM of each forward and reverse primer in the supplied SuperFI Buffer. PCR was done with the following program: Initial denaturation at 98°C for 3 min, 25 cycles of amplification (98°C for 30 s, 62°C for 20 s, 72°C for 2 min) and a final elongation at 72°C for 5 min. The forward and reverse primers used include custom 24 nt barcode sequences followed by the sequences targeting abeV48A: (515FB) GTGYCAGCMGCCGCGGTAA and (1391R) GACGGGCGGTGWGTRCA (Apprill et al., 2015; Parada et al., 2016).

The resulting amplicon libraries were purified using the standard protocol for CleanNGS SPRI beads (CleanNA, NL) with a bead to sample ratio of 3:5. DNA was eluted in 25 μL of nuclease free water (Qiagen, Germany). Sequencing libraries were prepared from the purified amplicon libraries using the SQKLSK114 kit (Oxford Nanopore Technologies, UK) according to manufacturer protocol with the following modifications: 500 ng total DNA was used as input, and CleanNGS SPRI beads for library cleanup steps. DNA concentration was measured using Qubit dsDNA HS Assay kit (Thermo Fisher Scientific, USA). Gel electrophoresis using Tapestation 2200 and D1000/High sensitivity D1000 screentapes (Agilent, USA) was used to validate product size and purity of a subset of amplicon libraries.

The resulting sequencing library was loaded onto a MinION R10.4.1 flowcell and sequenced using the MinKNOW 22.12.7 software (Oxford Nanopore Technologies, UK). Reads were basecalled and demultiplexed with MinKNOW guppy g6.4.2 using the super accurate basecalling algorithm (config r10.4.1_400bps_sup.cfg) and custom barcodes.

The sequencing reads in the demultiplexed and basecalled fastq files were filtered for length (320–2000 bp) and quality (phred score > 15) using a local implementation of filtlong v0.2.1 with the settings –min_length 320 –max_length 2000 –min_mean_q 97. The SILVA 16S/18S rRNA 138 SSURef NR99 full length database in RESCRIPt format was downloaded from the QIIME on 29 September 2022 (Yilmaz et al., 2014; Robeson et al., 2021). Potential generic place holders and deadend taxonomic entries were cleared from the taxonomy flat file, i.e., entries containing uncultured, metagenome or unassigned, were replaced with a blank entry. The filtered reads were mapped to the SILVA 138.1 99% NR database with minimap2 v2.24r1122 using the axmapont command (Li, 2018) and downstream processing using samtools v1.14 (Danecek et al., 2021). Mapping results were filtered such that query sequence length relative to alignment length deviated < 5 %. As a data denoising step, OTUs with low abundance, constituting less than 0.01% of the total mapped reads within each sample, were disregarded. Further bioinformatic processing was done via RStudio IDE (2022.2.3.492) running R version 4.2.3 (20230315) and using the R packages: for conveniently analyzing 16S rRNA amplicon data, the Ampvis2 (2.7.27) (Albertsen et al., 2015) package was used, while the iNEXT (2.0.20) package was utilized for diversity calculations. The ShortReads (1.54.0) package was employed for quality control and trimming. Lastly, the Seqinr package (4.2.16) was used for the visualization of biological sequences (Chao et al., 2014; Hsieh et al., 2016). Sequencing dataset is available through the Sequence Read Archive (SRA) under accession PRJNA1039605.

### 2.4 Bacterial isolation, identification, and mineral experiments

Culturable bacteria isolation: a fragment of microbialite from the site 2 was sampled using a sterile spatula in a sterile 15 ml Falcon tube and stored at 4°C until the return to lab. A total 3 ml of sterile distilled water were added, the microbialite was crushed with a steril stub and vortexed. The suspension was diluted 10^3^ times with sterile distilled water and 0,1 ml were spread onto Lysogeny Broth (LB) pH9 plates (1%Tryptone, 1% NaCl, 0,5% Yeast Extract, 2% BactoAgar, pH was adjusted to 9 with NaOH), incubated at 28°C for 4 days. Growing colonies were observed and, based on different morphology/morphotype, strains were streaked and then isolated on LB pH9 plates.

We selected 8 isolates that showed different colony morphology and we called them with progressive numbers 3bis1 to 3bis8. The isolates were grown in liquid LB pH 9 to saturation then 0,8 ml of culture were mixed with 0,4 ml 60% glycerol to get a 20% glycerol final concentration before being stored at −80°C to establish a culture bank. The selected isolates 3bis2, 3bis5, 3bis7 and 3bis8 were characterized at molecular level. DNA extraction from a single colony was carried out using microLYSIS buffer (Labogen, Rho, Italy) following the manufacturer's instructions and 2 μl of genomic DNA were used for the amplification of partial 16S ribosomal RNA genes. PCR was performed using the universal bacterial primers P0 (5'-GAGAGTTTGATCCTGGCT-3') and P6 (5'CTACGGCTACCTTGTTAC-3') as described in Ventura et al. (2000). The 16S ribosomal RNA gene amplification was verified by agarose gel electrophoresis. The band of about 1500 bp was purified (Gel/PCR DNA fragments Extraction kit Geneaid cat. DF 100) and sequenced with both primers by Sanger method by a sequencing service (http://www.biofabresearch.it/index2.html). All the resulting sequences were analyzed using the online BLAST nucleotide tool, available at: https://blast.ncbi.nlm.nih.gov.

Selected bacterial strains were tested for calcium carbonate production by multiple dropping (15–30 μl) of cultures grown to saturation in liquid LB pH9 onto solid B4 precipitation growth medium. B4 agar plates were prepared accordingly to Marvasi and colleagues (2011), with the following modifications: 0.4% yeast extract (w/v), 0.5% dextrose (w/v), and 1.4% agar (w/v) (final concentrations). After autoclaving, 0.3% (w/v, final concentration) of urea, sterilized by filtration, and calcium chloride (0.25% w/v, hereafter named B4CaCl_2_) or calcium lactate (0.25% w/v, hereafter named B4CaLact) were added. B4 pH was around 6.0 and was adjusted to 8.0 with 10N NaOH (Marvasi et al., 2012). Moreover, additional B4 agar plates were prepared using 0.22 μm filtered lake water supplemented by 0.4% yeast extract, 0.5% dextrose, and 0.3% urea (B4LW), added to 5% autoclaved Bacto agar to get a final agar concentration of 1.5% Agar. In these plates, lake water was about 70% (v/v) of total.

Cultures of selected strains (15–30 μl) were spotted onto B4CaCl_2_, B4CaLact, and B4LW plates, incubated at 28°C and examined periodically at the indicated days after inoculation by optical and fluorescence microscopy Axioskop2 (Carl Zeiss, Jena, Germany), objective 5X, equipped with a digital camera (micro-CCD) or Carl Zeiss stereomicroscope for the presence of crystals. Control plates without bacterial inoculation and incubated in the same conditions, did not produce any precipitation, nor were present crystals outside of the site of bacterial inoculation (Supplementary Figure SM2).

For crystal analysis, they were removed from the colonies on plates by a spatula and resuspended in 1 ml distilled water in 1.5 ml tubes. Tubes were centrifugated at 1,000 rpm (94 RCF) for 2 min and supernatant containing bacterial cells was removed. This step was repeated until the supernatant was clear. Crystals were allowed to air-dry at 37°C prior to SEM and X-ray diffraction analysis, as described above. Concerning hazenite crystals showed in Figure 6, they were removed with a needle from the plates and put directly on the stub for SEM analysis.

### 2.5 Bacterial cell visualization

In order to visualize bacteria within the original microbialites fragments, Catalyzed Reported Deposition-Fluorescence *in situ* Hybridization (CARD-FISH) was applied as described previously (Lupini et al., 2011; Fazi et al., 2021). A specific rRNA-target Horseradish peroxidase labeled oligonucleotidic probe (LGC354a, Biomers, Ulm, Germany) targeted Bacteria and various genera within the class Bacilli (phylum Firmicutes), e.g., *Bacillus, Exiguobacterium, Leuconostoc, Weissella* or *Lactobacillus* (Meier et al., 1999; Amalfitano and Fazi, 2008). Cells were then stained with DAPI solution. The stained microbialites fragments were then observed under a confocal laser scanning microscope (CSLM; Olympus FV1000) at a magnification of 60x. Both DAPI stained cells were excited by 405 nm light and emitted at 430 to 470 nm (blue color). The hybridized bacterial cells were excited with the 488 nm line of an Ar laser (excitation) and observed in the green channel from 500 to 530 nm (emission). Precipitated minerals were visualized by their reflection signal (405 nm line of a diode laser) and appear of gray color (Venturi et al., 2022). The three-dimensional reconstruction of CSLM images was elaborated by the software IMARIS 7.6 (Bitplane, Switzerland) with 3D volume rendering mode.

## 3 Results

### 3.1 Minero-petrographic characteristics of the microbialites

**Figure 2:**
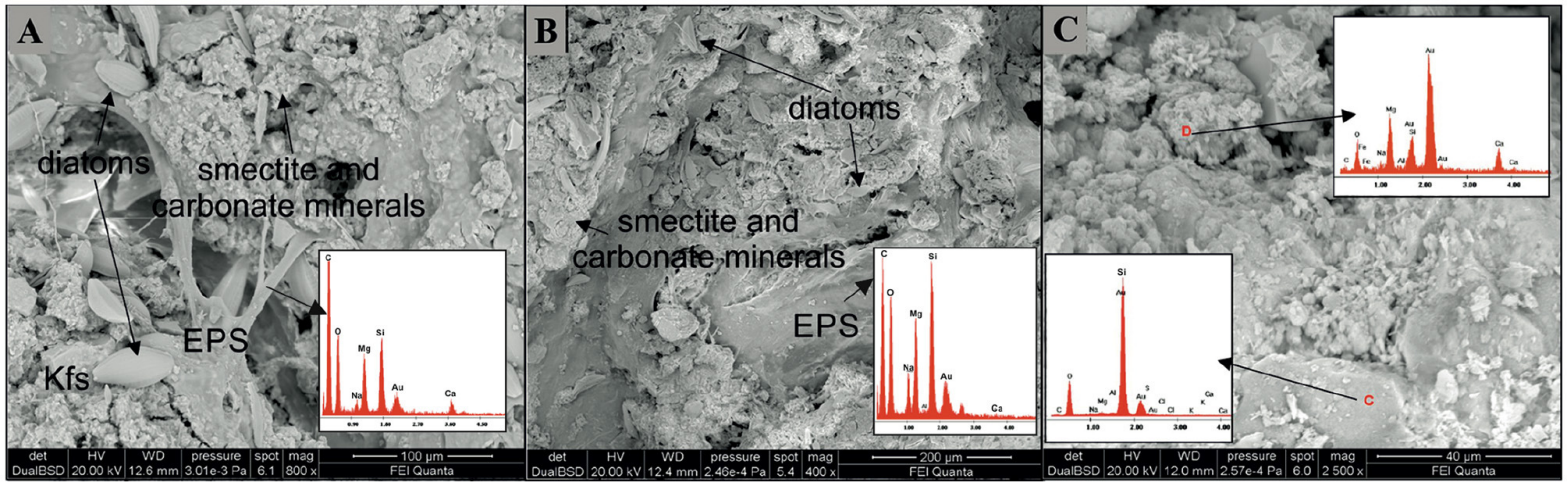
SEM images representative of the three differently colored layers. **(A)** In the W layer a dense EPS network with abundant diatom frustules and aggregates of silicate (Mg-smectite) and carbonate minerals is observable. **(A)** detrital feldspar occurs in the lower left. **(B)** In the G layer the EPS matrix in association with smectite aggregates is diffuse. Diatom frustules, many of them partially corroded are scarcely represented. **(C)** In the B layer aggregates of carbonate minerals along with smectite coat detrital minerals. Note in the red circle the presence of rosette-like clusters of calcium-carbonate minerals (possibly aragonite). Kfs, K-feldspar; EPS, Extracellular Polymeric Substances.

The mineral assemblage resulting from XRD analyses of microbialites consisted mostly of carbonate minerals (aragonite, calcite, monohydrocalcite and hydromagnesite; 28%−45%) and feldspars (K-feldspar and anorthoclase; 46%−63%) and subordinate amounts of smectite (4%−9%) and quartz (3%−5%) (Table 1). The SEM analyses confirm the mineral phases observed by XRD (Figure 2) i.e., carbonate, detrital minerals (quartz and feldspars) and clay mineral (smectite). The W layer is characterized by high concentration of diatoms enveloped by a film of EPS (Figure 2A) as well as by detrital crystals (feldspar, Figure 2A) and calcium carbonates occasionally coated by aggregates of smectite. In the G layer (Figure 2B) EPS biofilm appears more widespread, while diatom frustules are scarce and frequently show signs of dissolution. Also in this sample some EDS spectra are compatible with the composition of carbonate and smectite minerals. SEM images of B layer does not show the presence of diatoms or EPS matter but in it are recognizable carbonate crystals (Figure 2C) along with smectite covering detrital quartz and feldspar.

**Table 1:** Representative mineral assemblages (wt %) forming the microbialites (sample W, G, and B layers) (XRD).

**Sample ID**	**Whole rock composition (wt %)**							
	**Qz**	**Cal**	**Arg**	**Hmg**	**MhCal**	**Sm**	**Kfs**	**An**
W	3	9	21	3	6	9	20	29
G	5	13	29	3	-	4	21	25
B	4	9	18	1	-	5	22	41

*Qz, quartz; Cal, calcite; Arg, aragonite; Hmg, hydromagnesite; MhCal, monohydrocalcite; Sm, smectite; Kfs, K-feldspar; An, anorthoclase*.

### 3.2 Microbial community composition

**Figure 3:**
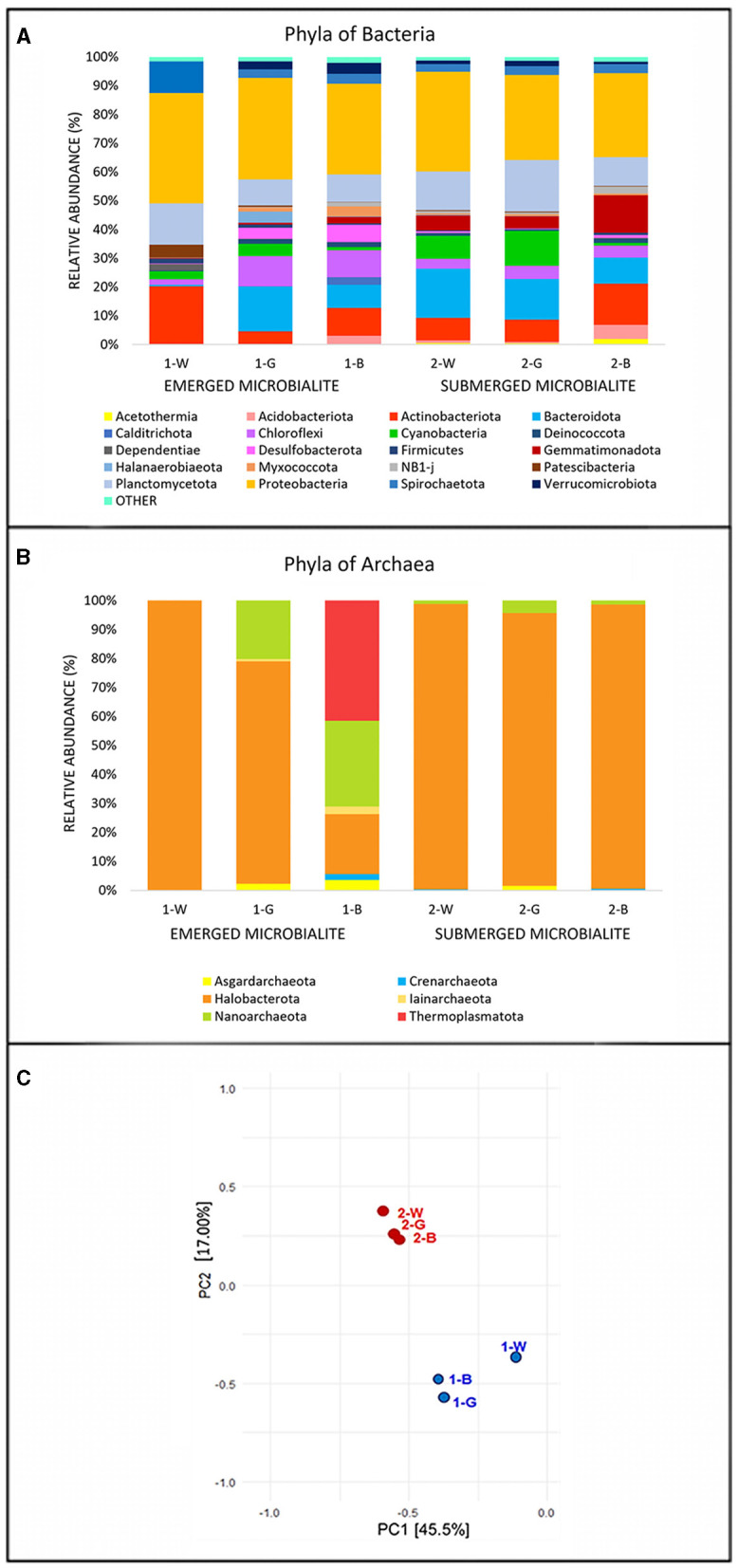
Relative abundance (%) of the most prevalent phyla of **(A)** Bacteria and **(B)** Archaea. The graph shows only phyla which contributed more than 1.5% to the total bacterial community in at least one sample. The abundance of the remaining phyla was summed and labeled as “other”. **(C)** Principal Component Analysis (PCA) performed on the phyla of Bacteria and Archaea. Identification of samples with similar microbial communities using PCA. Each point represents the microbial community in each specific sample. The distance between points on the plot corresponds to similarity; closer points refer to samples with highly similar microbial communities (source: Rstudio, version 4.3.1).

The microbialite samples exhibited similar relative abundances across the major phyla, with slight variations specific for each sample. The Bacteria domain (Figure 3A) was primarily composed of Proteobacteria (average abundance of 33.1%). Notably, the classes *Alphaproteobacteria* and *Gammaproteobacteria* were more represented in the most superficial (1-W, 2-W) and deepest layers of both sites (1-B, and 2-B). In the 1-W sample, the Alphaproteobacteria class was mainly represented by orders of *Rhodobacterales* (comprising 63.21% of the total proteobacteria) and *Holosporales*. Conversely, *Defluviicoccales* and *Tristellales* orders are more abundant in the all remaining samples (both absent in 1-W). Within the class *Gammaproteobacteria, Coxiellales* and *Pseudomonadales* orders are exclusive to 1-W, while *Ectothiorhodospirales* and *Steroidobacterales* orders are more abundant in the other microbialite samples. Notably, the most pronounced differences are evident solely in the emerged superficial microbialite sample, 1-W. In contrast, across all other samples, including the submerged microbialite specimens, orders and families exhibit constant patterns, with slight fluctuations in relative abundance.

The phylum *Actinobacteriota*, displayed an average abundance of 10%, represented by the classes *Acidimicrobia*, showing the highest abundance of in the surface of the emerged microbialites (1-W 14.7%) and the class *Actinobacteria* in the deepest layer of the submerged (2-B 12.56%). The phylum Planctomycetota is present with an average abundance of 12.39% in all the microbialite samples, with maximum values corresponding to the samples 2-G (17%) and 1-W (15%), and minimum values in 1-G (9%) and 1-B (9%) samples. At the class level, there are differences among the various samples, for example the class *Planctomycetes* is exclusively present, with the *Pirellulaceae* family, in the surface sample of the emerged microbialite (100% in 1-W sample), while in the other samples the class *Phycisphaerae* prevails, especially in the submerged microbialite samples 2-W (85%) and 2-G (76%).

The Chloroflexi phylum shows an average of 10% among the emerged microbialite samples 1-G and 1-B, while in the superficial white layer of the same microbialite (1-W), this phylum is only present at 1.98%. In contrast, in the submerged microbialite samples, the relative percentage of this phylum tends to decrease, with an overall average of 4% across the three samples. The predominant classes are *Anaerolineae*, present in all samples, and *Chloroflexia* class (*Chloroflexaceae* family), which is present at 4.69% in sample 1-G and has an average of 1% in all the other samples. Interestingly, the Cyanobacteria phylum reaches its maximum expression in terms of percent relative abundance in sample 2-G, in the intermediate green layer of the submerged microbialite (12%), and in the submerged surface sample 2-W (7.96%), while in the emerged microbialite, Cyanobacteria show an average of 2.65%, The most substantial class of this phylum is *Cyanobacteria*, orders *Thermosynechococcales* with the family *Thermosynechococcaceae* (genus *Synechococcus IR11*), and the order *Synechococcales* with the family *Cyanobiaceae*. In the submerged microbialite samples, in addition to the aforementioned cyanobacterial orders, significant relative abundances are also present for the orders *Eurycoccales* and *Phormidesmiales* (*Nodosilineaceae* family). Conversely, in the emerged microbialite, the predominant order is C*yanobacteriales*, with the families *Microcystaceae* and *Xenococcaceae*.

The Archaea domain (Figure 3B) is represented on average by 81% for the phylum Halobacterota with the classes *Halobacteria* and *Methanosarcina* present in all sequenced samples. The phylum Thermoplasmatota is present only in the deepest layer of emerged microbiltes (1-B 41%), while the phylum Nanoarchaeota (class *Nanoarchaeia*, family *Woesearchaeales*) is present in the emerged microbialite samples (29% in 1-B and 20.1% in 1-G). Interestingly, the lowest number of reads, which is 20, recorded for the archaea domain in the sequenced samples is in the surface of emerged samples (1-W), predominantly attributed to the phylum Halobacterota, specifically the genera *Natronoarchaeum* and *Natronorubrum*.

In Table 2, a summary of eukaryotic sequences abundance, clearly highlights a higher presence of the eukaryotic kingdom in the submerged microbialite samples compared to the emerged ones. Gene sequencing of the 18S rRNA gene for the Eukaryota domain showed, based on the number of reads, the presence of Fungi, Protists, Algae, and other taxa. In particular, the phyla Ascomycota (genera *Aspergillus* and *Peniciullum*) and Basidiomycota (genus Malassezia) were found only in the upper layer of the emerged sample (1-W), while the phyla Amoebozoa, Cercozoa, and Apicomplexa have the highest number of reads in all submerged microbialite samples.

**Table 2:** Phyla of the Eukaryota domain present in samples of emerged microbialite (abbreviated as 1-W; 1-G, 1-B) and submerged (2-W, 2-G, 2-B), the symbols refer to the abundance of reads obtained from gene sequencing (18S rRNA) (-)1-15; (o) 16-50; (oo) 51-100; (^*^)101-150; (^**^>150).

**Sample ID**	**Fungi**		**Protist**			**Micro Algae**	**Green Algae**	**Red Algae**	**Animals**		
	**Asc**	**Bas**	**Amo**	**Api**	**Cer**	**Dia**	**Chl**	**Och**	**Pla**	**Rot**	**Nem**
1-W	o	o							-		-
1-G	o		o								^**^
1-B						oo					
2-W	-		oo	^*^	oo		oo	o		o	^**^
2-G			o	o					-		o
2-B			oo								o

*Asc, Ascomycota; Bas, Basidiomucota; Amo, Amoebozoa; Api, Apicomplexa; Cer, Cercozoa; Dia, Diatomea; Chl, Chlorophyta; Och, Ochrophyta; Pla, Platyhelminthes; Rot, Rotifera; Nem, Nematozoa*.

Regarding micro algae, their ssu rRNA genes were absent in the upper layers of the emerged microbialite and are only present in sample 2-W (submerged surface), with the phyla Chlorophyta, Ochrophyta, and Diatomea (the latter is also present in sample 1-B with a reads count of 54 referred to *Bacillariophyceae* class). The highest number of reads associated with the Eukaryota domain is consistently attributed to the Nematozoa phylum, with a relative abundance of reads >150 in sample 1-G and in the superficial layer of submerged microbialite 2-W.

To assess the similarities in microbial communities among samples, Principal Component Analysis (PCA) was conducted based on 16S rRNA gene sequencing results. The analysis revealed (Figure 3C) distinct patterns of microbial diversity, showing the pronounced differences between emerged and submerged microbialite samples, with the upper layer of emerged samples (1-W) exhibiting the most distinctive composition.

### 3.3 Isolates growth and mineralization

Five out of the 8 isolates were characterized at molecular level by 16S amplification and sequencing. The bacterial isolate 3bis2 was determined to be *Stappia sp*., with a 96.68% genetic similarity with the *Stappia indica* strain ZJY-144. Similarly, bacterial isolate 3bis3 was identified as *Bacillus sp*., exhibiting a high identity of 99.38% with an isolated strain CAU 1668 of *B. solitudinis*. The isolates 3bis7 and 3bis8 were recognized as *Metabacillus niabensis* (96% identity) and *Bacillus cereus* (98% identity), respectively. Additionally, strain 3bis5 was confirmed as *Sutcliffiella horikoshii*, with a genetic similarity of 99.33%. GeneBank accession number and closest bacterial relatives for each strain are reported in Table 3.

**Table 3:** Identification of closest bacterial relatives of strains isolated from a fragment of the submerged microbialite at the site 2 based on 16S rRNA gene sequence.

**Isolates**	**GeneBank accession number**	**Closest bacterial relatives**	**Sequence ID**	**Identity**
3bis2	OR770211	*Stappia indica*	KP282738.1	96.68%
3bis3	OR770051	*Bacillus solitudinis*	OK664947.1	99.38%
3bis5	OR770470	*Sutcliffiella horikoshii*	LN650588.1	99.33%
3bis7	OR770473	*Metabacillus niabensis*	HQ234281.1	96%
3bis8	OR772953	*Bacillus cereus*	MG763124.1	98%

**Figure 4:**
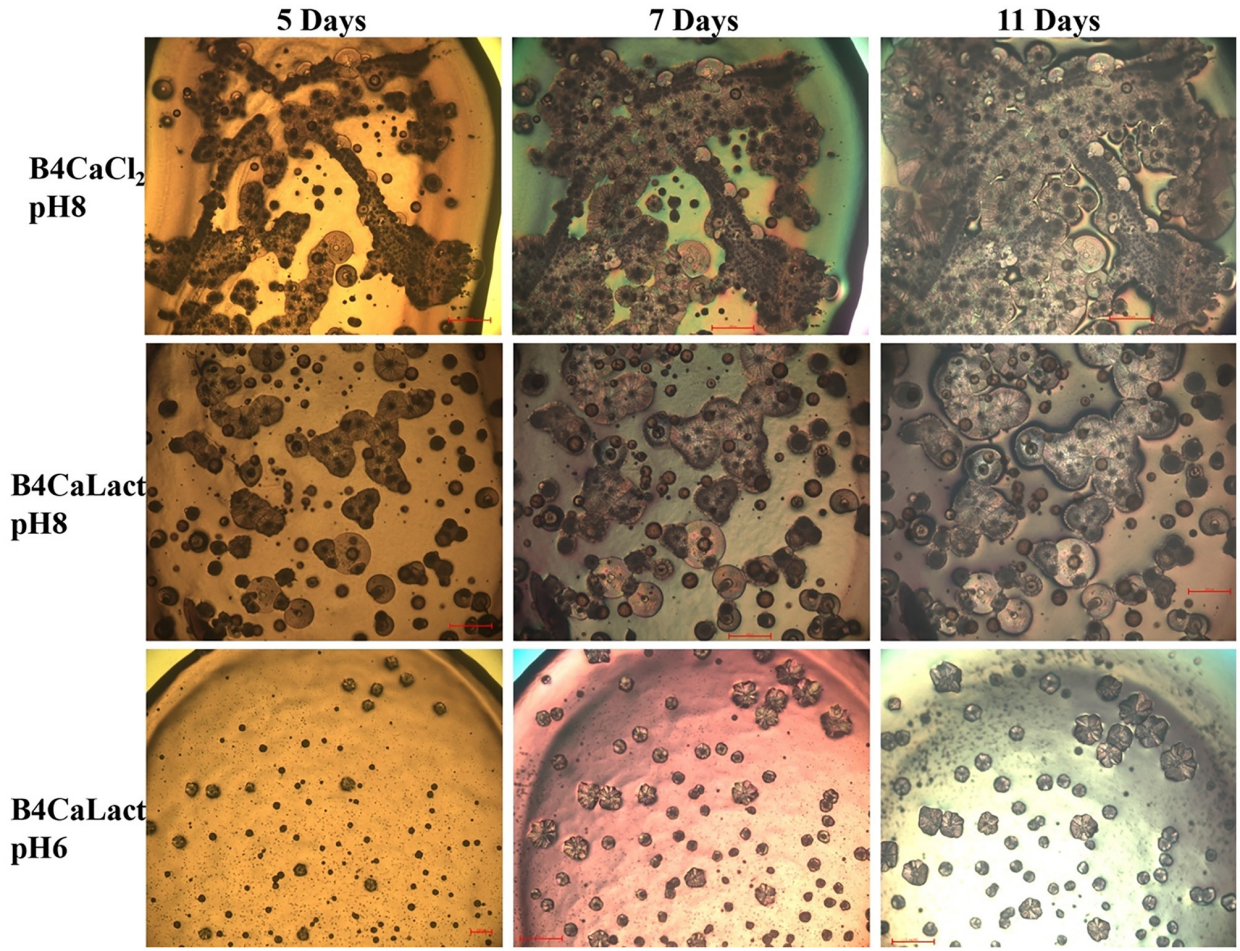
Crystal formation kinetics. Cells from saturated culture of *Stappia sp*. 3bis2 strain were spotted onto B4CaCl_2_ pH8, B4CaLact pH8 and B4CaLact pH6 plates, incubated at 28°C and observed by optical microscopy after 5, 7 and 11 days. Scale bar: 100 μm in B4CaLact pH6, 5 Days, 200 μm in all the other panels.

All these microbial isolates demonstrated the ability to induce Microbially Induced Calcium Carbonate Precipitation (MICP). As illustrated in Figure 4, the kinetic of crystal formation for the *Stappia sp*. 3bis2 strain on different B4 media was observed through optical microscopy. Crystal formation occurred within 2–5 days of incubation, with sizes increasing over time, irrespective of the calcium source supplied (CaCl_2_ or Ca-lactate), or initial pH. However, the appearance of crystals was notably slower when the initial pH was low (pH 6), in contrast to the condition at pH 8 (Figure 4).

**Figure 5:**
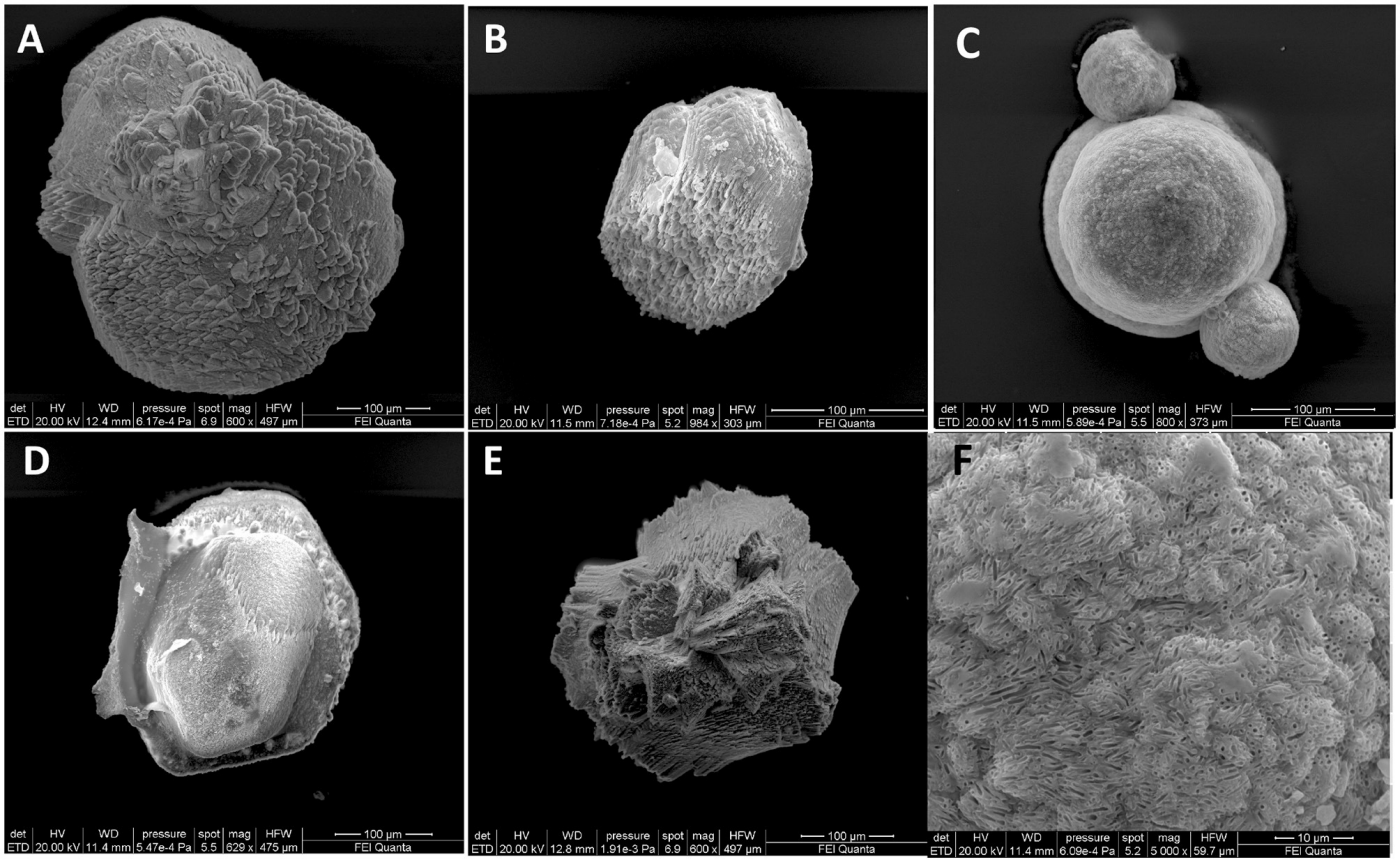
SEM images representative of carbonate-crystals from strains **(A)** *Stappia sp*. 3bis2; **(B)** *Bacillus sp*. 3bis3; **(C)** *Sutcliffiella sp*. 3bis5; **(D)** *Metabacillus sp*. 3bis7; **(E)** *Bacillus sp*. 3bis8 grown in B4CaLactate medium for 16 (3bis2) and 24 days (3bis3, 5, 7, and 8). **(F)** is an enlargement of **(C)** showing bacterial cells calcification.

As shown in Figure 5, all strains were capable of precipitating calcium carbonates although with different shapes. Figure 5F shows an enlargement of crystals produced by the *Sutcliffiella sp*. 3bis5 strain where the calcifications of the bacterial cells can be observed, indicating that precipitation took place around specific cells, which subsequently experienced lysis.

X-ray diffraction (XRD) analysis of precipitates showed only slight different mineral assemblages (Table 4) depending on the administered substance (CaCl_2_, Ca-lactate) and strain. The bacteria grown on plates with the addition of CaCl_2_ mostly formed calcite (84%−90%), vaterite (5%−11%) and ankerite (3%−5%) and subordinate amounts of iron oxide (maghemite). The bacteria supplied by Ca-lactate formed precipitates constituted by calcite (from 60% to 95%), ankerite (from 2 to 30%), very low amounts of vaterite (2%−3%) and maghemite (1%). Detrital minerals such as quartz and kaolinite have been identified in all the bacteria precipitates with contents that do not exceed 6% and 1% respectively.

**Table 4:** XRD analysis of precipitates by isolated strains. Media, initial pH, and incubation time (Days) are also indicated the recognized minerals (wt %) Qz-quartz, Cal-calcite, Arg-aragonite, Vtr-vaterite, Ank-ankerite, Mgh-maghemite, Hzn-Hazenite, Kln-Kaolinite.

**Strain**	**Media**	**pH**	**Days**	**Qz**	**Cal**	**Vtr**	**Ank**	**Mgh**	**Hzn**	**Kln**
*Stappia sp*. 3bis2	B4CaCl_2_	6	22	1	84	11	3	1	-	-
*Stappia sp*. 3bis2	B4CaCl_2_	8	13	1	90	3	5	1	-	-
*Stappia sp*. 3bis2	B4CaLact	6	14	1	90	-	8	1	-	-
*Stappia sp*. 3bis2	B4CaLact	8	16	1	93	3	2	1	-	-
*Bacillus sp*. 3bis3	B4CaLact	8	24	2	87	-	10	1	-	-
*Sutcliffiella sp*. 3bis5	B4CaLact	8	24	6	60	-	33	1	-	-
*Metabacillus sp*.3bis7	B4CaLact	8	24	6	64	-	29	1	-	-
*Bacillus sp*. 3bis8	B4CaLact	8	24	1	95	-	3	1	-	-
*Stappia sp*. 3bis2	B4LW	9	35	-	7	-	6	-	86	1

**Figure 6:**
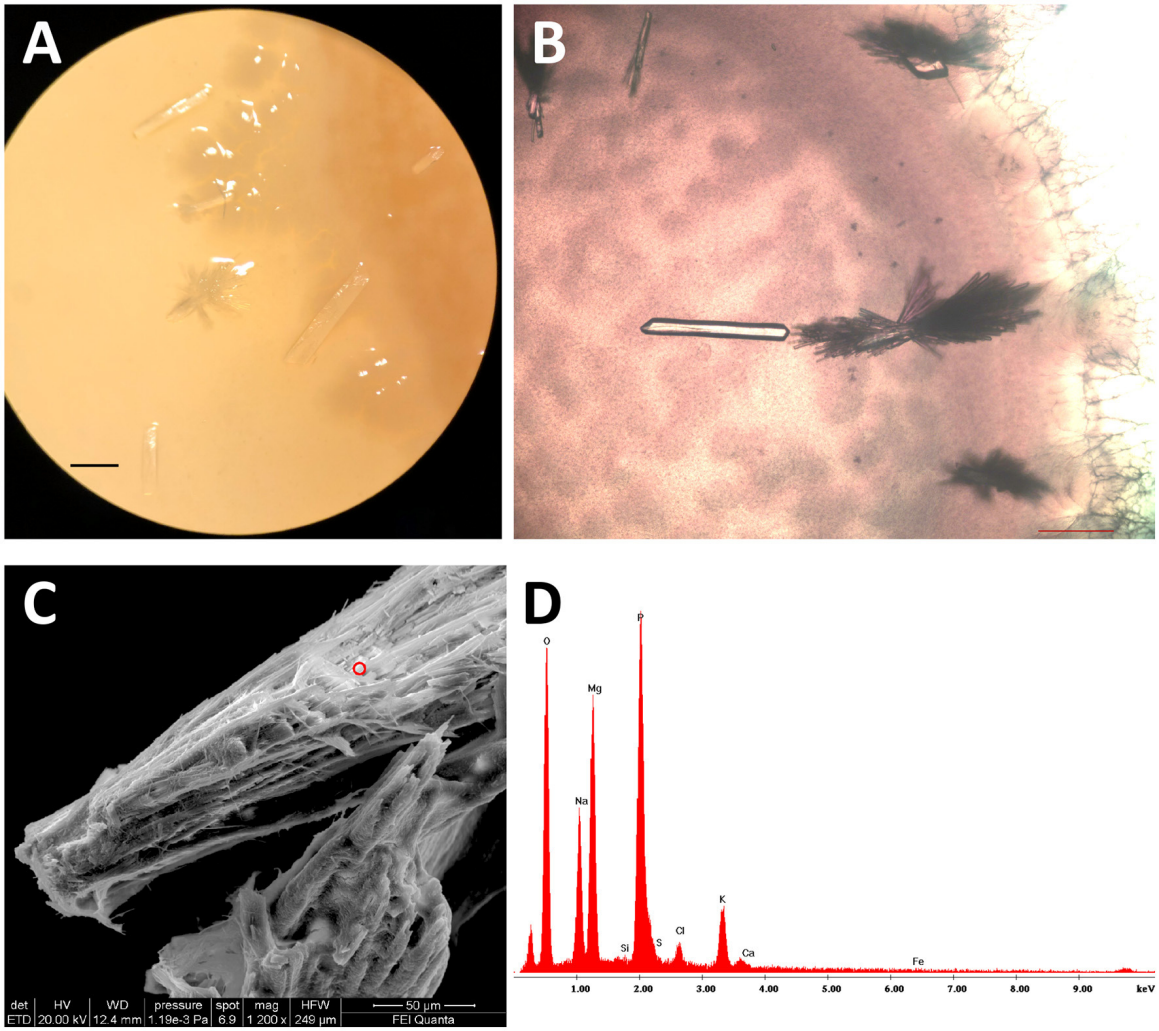
*Stappia sp*. 3bis2 cells formed hazenite when deposited onto B4LW and incubate for 35 days at 28°C. **(A)** Stereomicroscope image; scale bar: 1 mm; **(B)** Optical microscopy image showed crystals in the bar or aciculate forms; scale bar 200 μm. **(C)** Field-emission scanning electron microscopy (FE-SEM) of crystals, red circle represents the point where EDS was applied; **(D)** EDS.

**Figure 7:**
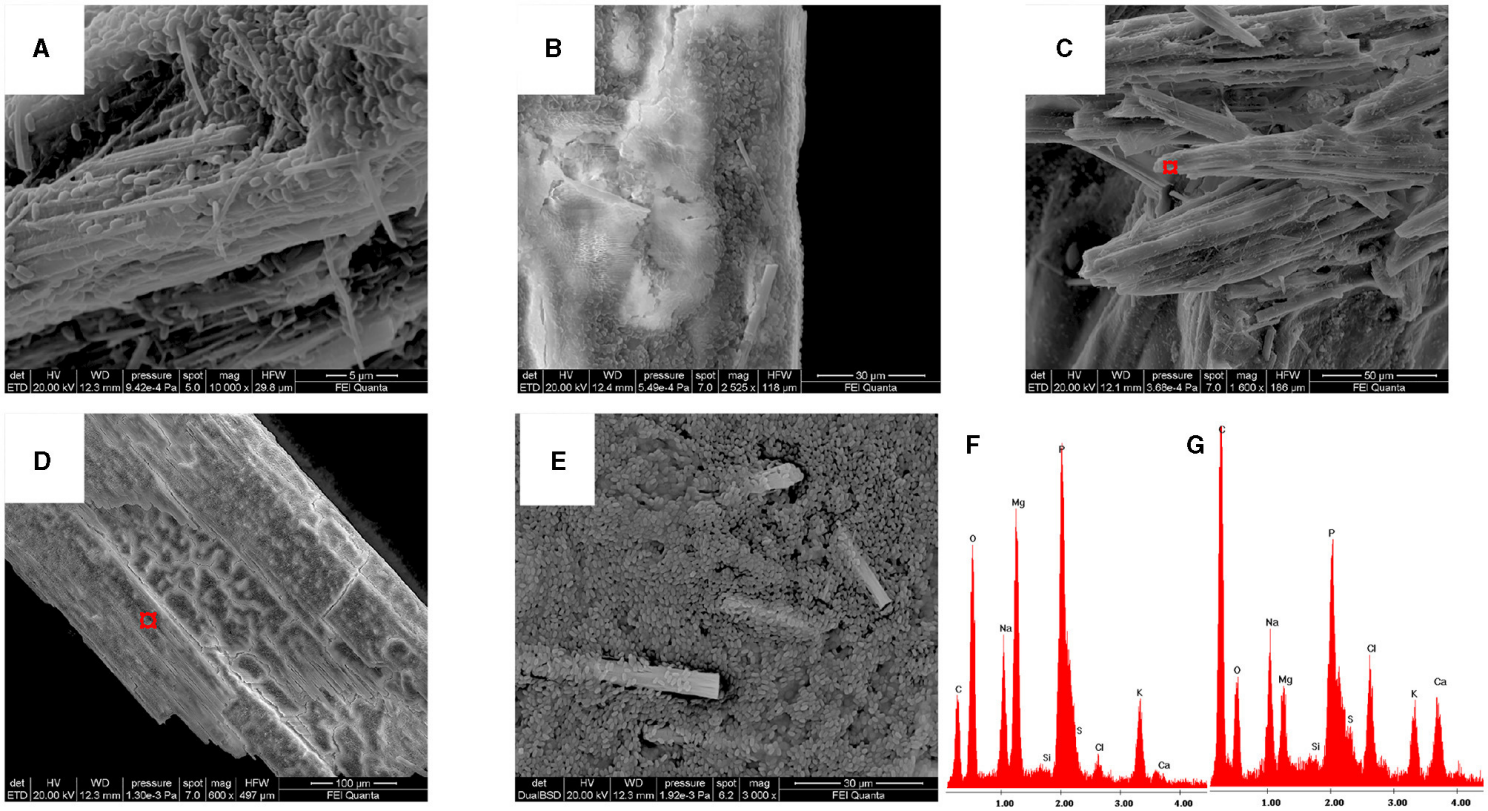
All the isolates can precipitate hazenite after 35 days of incubation. **(A)** *Stappia sp*. 3bis2; **(B)** *Bacillus sp*. 3bis3; **(C)** *Sutcliffiella sp*. 3bis5; **(D)** *Metabacillus sp*. 3bis7; grown in B4LW for 35 days (**B, C, D)**; **(E)** *Bacillus sp*. 3bis8 grown in B4LW for 100 days; **(F, G)** represent EDS analysis of regions indicated by the red squares in **(C, D)**, respectively.

All the isolates grown on B4LW media, containing 0.22 μm filtered Pantelleria lake water, precipitated crystals, which appear white/transparent with an aciculate or bar morphology at the stereomicroscope, optical and SEM microscope. As an example, crystals from *Stappia sp*. 3bis2 strain are shown in Figure 6. The EDS analysis revealed the occurrence of K, Na, Mg and phosphorous. The X-ray diffraction analysis performed on crystals obtained from the *Stappia sp*. 3bis2 strain, showed that these crystals were phosphate minerals such as hazenite [KNaMg_2_(PO_4_)_2_ · 14 H_2_O] that represent about 85% in mass of the sample, the rest was constituted by carbonates (calcite and ankerite) (Table 4, Supplementary Figure SM1). For SEM analysis, crystals were taken directly from the culture and deposited on a stub for SEM microscopy, for this reason bacterial cells were most of the time visible on the precipitated (Figures 7A–E). For *Bacillus sp*. 3bis8, crystals were collected after an extended 100-day incubation period (Figure 7E). In Figures 7F and G is reported EDS analysis relative to crystals shown in Figures 7C and D, respectively.

**Figure 8:**
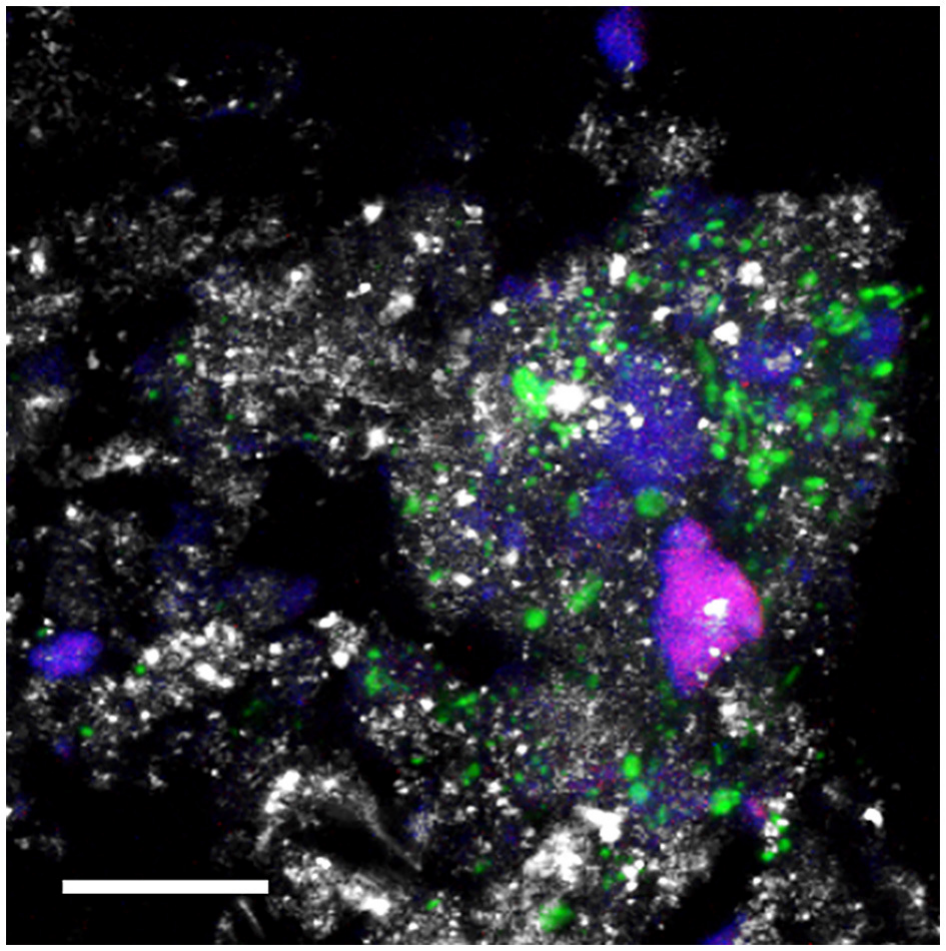
CLSM combined images showing the spatial distribution of Bacteria belonging to the class Bacilli (phylum Firmicutes (green) and other DAPI stained cells (blue) identified by CARD-FISH in the deep layers of the natural microbialite (1-B). Autofluorescent cells appear in red. The hybridized bacterial cells were excited with the 488 nm line of an Ar laser (excitation) and observed in the green channel from 500 to 530 nm (emission). Mineral crystals were visualized by their reflection signal (405 nm line of a diode laser) and appear of gray color. Bar = 20 μm.

The confocal microscope visualization (Figure 8) of microbialite samples unequivocally highlights the presence of Firmicutes labeled with the LGC354a probe. In Figure 8, the submerged microbialite sample is depicted, specifically the superficial layer 2-W, confirming the presence of bacterial cells belonging to the Firmicutes phylum associated to mineral granules. Additionally, cells labeled with the DAPI dye are highlighted in blue. Through the autofluorescence signal, it is also possible to observe the presence of non-filamentous cyanobacteria.

## 4 Discussion

Microbialites are organo-sedimentary rocks formed because of the vital actions of benthic microbial communities, particularly cyanobacteria and algae, which capture sediment and/or induce the precipitation of carbonate minerals (Moore and Burne, 1987). The significance of these bio-sedimentary processes in carbonate production has long been recognized throughout the geological record (Riding, 2006).

In this study, the bioprecipitation of carbonate and non-carbonate minerals were investigated in two microbialites under different water availability (emerged *vs* submerged). The superficial layer of the emerged microbialite (1-W) exhibited a peculiar pattern of microbial diversity, as visualized by the PCA plot, being subjected to higher environmental variability (e.g., water availability, solar radiation, availability of nutrients). In this layer Actinobacteriota phylum represented the 20.27% of the total reads, and 10.89% is referred to the Verrucomicrobiota phylum, which is completely absent in the other samples. Several studies have highlighted the remarkable abilities of Actinobacteria to thrive in extreme conditions, including high pH, temperatures, and water stress. They are known for their ability to utilize a variety of substrates, including less degradable compounds such as chitin, cellulose, and hemicellulose, in addition to their resistance to UV radiation (Warnecke et al., 2007). Additionally, it has been shown that many members of the phylum Verrucomicrobiota are obligate aerobic heterotrophic bacteria (Cabello-Yeves et al., 2017), and therefore present only in the superficial part of the emerged microbialite, in direct contact with the terrestrial atmosphere. Beyond the Bacteria domain, substantial differences between the 1-W layer and all others are highlighted by the Eukaryote domain, which exhibits more terrestrial organisms. The phyla Ascomycota and Basidiomycota from the Fungi kingdom are present only in the 1-W. The species identified in this sample, *Aspergillus restrictus*, belonging to the *Aspergillaceae* family, is known to be a xerophilic species (Abdel-Azeem et al., 2016). Eukaryotes may play cohesive and/or calcifying roles to the structure of microbialites. For instance, in marine or brackish environments, filamentous algae associated with stromatolites can trap grains, foraminifera can stabilize particles (Riding, 2006), and eukaryotic algae can promote carbonate precipitation through their photosynthetic activity (Riding, 2000).

The sample clustering according to the PCA plot showed relatively high similarity among the two distinct groups of emerged and submerged microbialites. The submerged microbialite showed a higher similarity among the different layers, probably because of the more similar chemical and physical conditions they are exposed to. They exhibit high percentages of Proteobacteria, Bacteroidota, and Actinobacteriota, consistent with Saghaï's findings (2015). In our study, there is still a high percentage of sequences that do not show significant homology with any of the sequences in the SILVA 138.1 99% NR database. This could suggest that they represent what is currently termed the dark microbiome, to refer to the community of microorganisms whose phylogenetic identity has not yet been determined, but which are detected using high-throughput methods such as direct DNA sequencing (Marcy et al., 2007; Azua-Bustos et al., 2023). For instance, the class *Gammaproteobacteria* exhibits an unknown reads percentage of 24% in the intermediate layer of the submerged microbialite (2-G). The redundancy of taxa across different microbialites studied in various locations around the world, such as in the study conducted the alkaline Lake Van, Turkey (López-García et al., 2005) suggests their involvement in the biomineralization process and microbialite formation.

Furthermore, despite cyanobacteria being traditionally considered the primary drivers in the biomineralization process, even in the sampled intermediate layer characterized by a green color, they showed a relative abundance of only 4.24% in 1-G and 12% in 2-G. This finding aligns with the results obtained by Cangemi et al. (2016), as they did not detect cyanobacteria in the green portion of the microbialite collected in the same lake. It has been demonstrated that various metabolic processes promote carbonate supersaturation, including oxygenic and anoxygenic photosynthesis, as well as sulfate reduction and anaerobic methane oxidation coupled with sulfate reduction (Saghaï et al., 2015). Members of certain genera within the phylum Firmicutes, as an example, are sulfate reducers and could substantially contribute to carbonate precipitation (Bott, 2014). Among the most prevalent families of the phylum Firmicutes, we found the family *Dethiobacteraceae*. This family comprises obligatory anaerobic, moderately salt-tolerant, and obligatory alkaliphilic bacteria capable of chemolithoautotrophic growth through elemental sulfur disproportionation and fixing CO_2_ via the Wood–Ljungdahl pathway (Sorokin and Chernyh, 2017). Within the phylum Proteobacteria, belonging to the class *Gammaproteobacteria*, we identify the family *Alcanivoracaceae*, Gram-negative aerobic rod-shaped bacteria commonly isolated from marine waters and sediments worldwide, which includes many species that can utilize aromatic compounds (Silveira and Thompson, 2014); the *Ectothiorhodospiraceae* family, typically consists of halophilic and/or alkaliphilic purple sulfur bacteria that thrive under anaerobic conditions in the presence of light, using reduced sulfur compounds as photosynthetic electron donors (Imhoff et al., 2019). While their primary metabolic mode is photoautotrophic with the deposition of elemental sulfur globules outside the cell, some species can also grow photoheterotrophically; the family *Chromatiaceae* (highly abundant in samples 1-G and 1-B) is the main family of purple sulfur bacteria (PSB), anoxygenic phototrophic *Gammaproteobacteria* that utilize sulfide as an electron source for carbon fixation (Thiel et al., 2018). The class *Alphaproteobacteria* is mainly represented by the family *Rhodobacteraceae*, known for both oxygenic and anoxygenic photosynthesis (Imhoff et al., 2019). The phylum Chloroflexi includes a varied array of organisms, ranging from anoxygenic photoautotrophs to aerobic chemoheterotrophs, thermophiles, and anaerobic organisms utilizing reductive dehalogenation of organic chlorinated compounds for energy acquisition (Gupta, 2013). In our study the main classes belonging to this phylum are *Chloroflexia* (*Thermomicrobiaceae* and *Chloroflexiaceae* families) which includes organisms that perform anoxygenic photosynthesis (Pierson and Castenholz, 1992), and the *Dehalococcoidia* class, which includes anaerobic bacteria capable of sulfate reduction. Only in the samples 1-G and 1-B of the emerged microbialite there are the families *Methanosarcina* and *Thermoplasmata*, known for their ability to produce methane (Zinder and Mah, 1979). This suggests a lower availability of oxygen in these two layers. Among the families comprising aerobic heterotrophic bacteria involved in the degradation of organic matter in the emerged microbialite, we found the families *Haloferacaceae* (1-W and 1-G), *Pirellulaceae* (1-W), *Phycisphaeraceae* (1-G and 1-B), while in all the layers of the submerged microbialite, there are the families *Rhodothermaceae* and *Nitriliruptoraceae*, and *Thermoanaerobaculaceae* and *Puniceicoccaceae* families which degrade organic matter through the fermentation process (Cho et al., 2011) (Supplementary Figure 3).

In our culture-dependent study five isolates were characterized at molecular level and 16S sequence analysis revealed that the strain 3bis2 belongs to the genus *Stappia*, namely *Stappia indica*, while all the others belong to *Bacilla* genera. The genus *Stappia* belongs to the order *Rhizobiales/ Hyphomicrobiales*, according to RDP or NCBI taxonomic lineage. *Stappia sp*. has been described in association with various marine invertebrates and microalgae (Park et al., 2017) and it can form a biofilm for the association with diatoms, inhibiting or promoting their growth (Vuong et al., 2020; Nair et al., 2022). A wide range of metabolic capabilities have been recorded for this genus, including aerobic anoxygenic photoheterotrophic and chemoorganoheterotrophic metabolisms using a range of carbon compounds (Weber and King, 2007). The bacterial isolate designated as 3bis3 is most closely related to *Bacillus solitudinins*, an alkaliphilic and moderately halophilic *Bacillus* strain recently discovered in alkali soils in Nima County, Tibet, China (Liu et al., 2019). Analysis of the 16S rRNA sequence of isolate 3bis5 revealed its classification as *Sutcliffiella horikoshii*, a novel genus within the *Bacillus* clade characterized by differences in nine conserved signatures indels (CSIs) (Gupta et al., 2020). The closest bacterial relative to isolate 3bis7 is *Bacillus niabensis*, now classified under *Metabacillus* sp. strains. This genus has recently been expanded to include *Bacillus solitudinis*. Members of the *Metabacillus* genus have been isolated from various environments, including soil, hypersaline lakes, and marine coastal regions, distinguishing themselves from *Bacillus* by six CSIs (Patel and Gupta, 2020). The isolate 3bis8 exhibits ~98% identity to *Bacillus cereus*, a known active participant in biomineralization processes (Han et al., 2013). It is worth noting that *Bacillus* genera, thanks to the properties of their cell wall and the production of EPS, is described as one of the most efficient microorganisms capable of precipitating calcium carbonate (Kim et al., 2015).

Results from bacterial cultivation on different media, showed that the studied isolates were capable to induce calcium carbonate precipitation. The MICP process has two potential CO_2_ sources to enable calcite precipitation: (i) CO_2_ produced during ureolysis and respiration, and (ii) CO_2_ dissolution present in the air or from dissolved minerals. The study conducted by Okyay and Rodrigues (2015) demonstrated that some environmental microbial isolates are able to sequester not only the CO_2_ produced by their own microbial metabolism, but also a 10% (v/v) excess of CO_2_ present in the headspace of serum bottles, underscoring the importance of microbial isolates in Next-Gen Carbon Capture. Experimental studies have shown that diverse heterotrophic bacteria can mediate CaCO_3_ precipitation, including several alkaliphilic and halophilic species, and ureolytic bacilli, suggesting that they could have a predominant role in the formation and growth of microbialites (Hammes et al., 2003; Rodriguez-Navarro et al., 2003). In addition, CaCO_3_ precipitation can be mediated by bacilli spores, which accumulate Ca^2+^ and other divalent cations in their walls (Marquis and Shin, 1994), and by sulfate reducing bacteria under anoxic conditions inside microbialites. Notably, firmicutes exhibited a remarkably low abundance, and the *Stappia* genera were conspicuously absent in the metagenomic analysis. Cultivation has been able to pick up microorganisms from microbialite samples involved in the precipitation process, including bacteria that have not been recovered from sequencing. This discordance between molecular and culture testing is often observed both in clinical (Rhoads et al., 2012; Mahnic et al., 2021) and environmental studies (Tytgat et al., 2014). The latter authors working on environmental samples, found that while, not unexpectedly, 77.5% of genera recovered by pyrosequencing were not among the isolates, 25.6% of the genera picked up by cultivation were not detected by pyrosequencing. The disparity between community analysis and isolated strains could be attributed to the culturing that favors and certain bacteria strains, such as *Bacillus* (Kim et al., 2016). Nevertheless, CARD-FISH analysis, performed in order to confirm the presence of Firmicutes in the *in situ* microbialites, showed the presence of this phylum.

When bacterial strains were cultivated in media containing lake water instead of distilled water, they precipitated a phosphate mineral identified as hazenite. The formation of the hazenite mineral in Mono Lake, investigated by Yang et al. (2014), has been linked to the metabolic activity of cyanobacteria of the genus *Lyngbya* and occurs in the presence of carbonates (Yang et al., 2011). Hazenite has been observed for the second time at the Belmaco Cave site (La Palma, Spain) (Fernández-Palacios et al., 2023). Although earlier studies have suggested that cultures containing the bacterial strain *Virgibacillus sp* can prompt the precipitation of an ammonium analog of hazenite, specifically (NH4)NaMg_2_(PO4)_2_·14H__2__O (Yang et al., 2014), for as much as we know, this is the first report of hazenite synthesis with K and Na, mediated by heterotrophic bacteria under controlled laboratory conditions.

The isolated strains showed high activity for calcium carbonate and phosphate precipitation and represent promising candidates for ecofriendly industrial applications (Zhang et al., 2022; Baidya et al., 2023). Like struvite and K-struvite, hazenite could also be a potential phosphorus source for long-term release for fertilizer use (Watson et al., 2020; Raniro et al., 2022). On the other hand, hazenite precipitation could be used as a bioremediation of industrial phosphate-rich wastewaters. Interestingly, although calcium and magnesium carbonates has been widely found, hazenite has not been found, up to now, in the recent microbialites in Bagno dell'Acqua lake. A possible explanation lies in the solubility of this mineral which could precipitate in particular conditions when the concentration of phosphorus increases, and dissolve in water when the concentration of phosphorus decreases, for example following rainfall events (Yang et al., 2011). An alternative interpretation might be that *in situ*, numerous microorganisms engage in competition for phosphate uptake, considering that lake water is not particularly rich in inorganic phosphate (Cangemi et al., 2016). In periods of drought, the harsh conditions provide an opportunity only for the most well-adapted organisms to facilitate the deposition of hazenite. This scenario seems plausible considering that hazenite was discovered for the first time in dry environment (Yang et al., 2011). However, the molecular mechanisms responsible for the biological deposition of hazenite remain unknown and require further investigation.

In conclusion, the analysis of the microbial diversity clearly demonstrate the dominance of non-photosynthetic microorganisms within the top layers of recent microbialites. Although it was possible to identify a common core-microbiome, at the surface the emerged microbialites hosted a different eukaryotic and prokaryotic community in comparison with those present in the submerged one. These results could help to better understand how the delicate equilibrium within the microbialites microenvironment could respond to the water level variability and therefore to the impact of climate changes. Moreover, our study clearly demonstrates the role of the microorganisms in mediating calcium carbon precipitations, parallel to PO_4_ precipitation. The results could bring new potential biotechnological applications for Next-Gen Carbon Capture and contemporaneous P recovery from P depleted environments.

## Data availability statement

The datasets presented in this study can be found in online repositories. The names of the repository/repositories and accession number(s) can be found at: https://www.ncbi.nlm.nih.gov/sra, PRJNA1039605.

## Author contributions

CM: Conceptualization, Data curation, Formal analysis, Investigation, Supervision, Writing—original draft, Writing—review & editing, Methodology. AP: Data curation, Formal analysis, Investigation, Writing—review & editing. LD: Investigation, Writing—review & editing. LA: Investigation, Writing—review & editing. CP: Validation, Writing—review & editing. AC: Validation, Writing—review & editing. MI: Investigation, Validation, Writing—review & editing. TR: Investigation, Writing – review & editing. AB: Investigation, Validation, Writing—review & editing. BC: Investigation, Validation, Writing—review & editing. FF: Project administration, Writing—review & editing. FC: Funding acquisition, Resources, Writing—review & editing. SF: Data curation, Formal analysis, Investigation, Methodology, Supervision, Writing—original draft, Writing—review & editing.

## References

[B1] Abdel-Azeem A. M. Salem F. M. Abdel-Azeem M. A. Nafady N. A. Mohesien M. T. Soliman E. A. (2016). “Biodiversity of the genus Aspergillus in different habitats,” in New and Future Developments in Microbial Biotechnology and Bioengineering (Amsterdam: Elsevier), 3–28.

[B2] Albertsen M. Karst S. M. Ziegler A. S. Kirkegaard R. H. Nielsen P. H. (2015). Back to basics–the influence of DNA extraction and primer choice on phylogenetic analysis of activated sludge communities. PLoS ONE 10:e0132783. 10.1371/journal.pone.0132783 26182345 PMC4504704

[B3] Amalfitano S. Fazi S. (2008). Recovery and quantification of bacterial cells associated with streambed sediments. J. Microbiol. Methods 75, 237–243. 10.1016/j.mimet.2008.06.004 18602952

[B4] Apprill A. McNally S. Parsons R. Weber L. (2015). Minor revision to V4 region SSU rRNA 806R gene primer greatly increases detection of SAR11 bacterioplankton. Aquatic Microb. Ecol. 75, 129–137. 10.3354/ame01753

[B5] Azua-Bustos A. Fairén A. G. González-Silva C. Prieto-Ballesteros O. Carrizo D. Sánchez-García L. Rampe E. (2023). Dark microbiome and extremely low organics in Atacama fossil delta unveil Mars life detection limits. Nat. Commun. 14:808. 10.1038/s41467-023-36172-1 36810853 PMC9944251

[B6] Baidya P. Dahal B. K. Pandit A. Joshi D. R. (2023). Bacteria-induced calcite precipitation for engineering and environmental applications. Adv. Mat. Sci. Eng. 2023:209. 10.1155/2023/261320934794448

[B7] Bajpai S. Shreyash N. Singh S. Memon A. R. Sonker M. Tiwary S. K. Biswas S. (2022). Opportunities, challenges and the way ahead for carbon capture, utilization and sequestration (CCUS) by the hydrocarbon industry: towards a sustainable future. Energ. Rep. 8, 15595–15616. 10.1016/j.egyr.2022.11.023

[B8] Baliva A. Marinangeli L. Piluso E. Ori G.-G. Ruscito V. (1999). Minero-Petrographic Studies of a Possible Martian Analogue Environment: Specchio di Venere Lake, Pantelleria Island (Italy). American Astronomical Society.

[B9] Bayoumy M. Abdallah A. M. Alam El-Din K. Nagy H. Shaltout M. (2019). Inter-annual variability and trends of sea level and sea surface temperature in the Mediterranean Sea over the last 25 years. Pure Appl. Geophys. 176, 3787–3810. 10.1007/s00024-019-02156-w

[B10] Bischoff K. Sirantoine E. Wilson M. E. George A. D. Mendes Monteiro J. Saunders M. (2020). Spherulitic microbialites from modern hypersaline lakes, Rottnest Island, Western Australia. Geobiology 18, 725–741. 10.1111/gbi.12400 32463178

[B11] Bott R. (2014). Brock Biology of Microorganisms, 14th Edn- Madigan. New York, NY: Igarss.

[B12] Cabello-Yeves P. J. Ghai R. Mehrshad M. Picazo A. Camacho A. Rodriguez-Valera F. (2017). Reconstruction of diverse verrucomicrobial genomes from metagenome datasets of freshwater reservoirs. Front. Microbiol. 8:303740. 10.3389/fmicb.2017.02131 29163419 PMC5673642

[B13] Cangemi M. Censi P. Reimer A. D'Alessandro W. Hause-Reitner D. Madonia P. Reitner J. (2016). Carbonate precipitation in the alkaline lake Specchio di Venere (Pantelleria Island, Italy) and the possible role of microbial mats. Appl. Geochem. 67, 168–176. 10.1016/j.apgeochem.2016.02.012

[B14] Chao A. Gotelli N. J. Hsieh T. C. Sander E. L. Ma K. H. Colwell R. K. Ellison A. M. (2014). Rarefaction and extrapolation with Hill numbers: a framework for sampling and estimation in species diversity studies. Ecol. Monogr. 84, 45–67. 10.1890/13-0133.137246386

[B15] Chen X. Zhang D. Larson S. L. Ballard J. H. Knotek-Smith H. M. Nie J. Han F. X. (2021). Microbially induced carbonate precipitation techniques for the remediation of heavy metal and trace element–polluted soils and water. Water Air Soil Pollut. 232:268. 10.1007/s11270-021-05206-z

[B16] Cho J. Yoon J. Hedlund B. P. (2011). Family I. Puniceicoccaceae. Bergey's Manual Syst. Bacteriol. 4:824. 10.1002/9781118960608.fbm00255

[B17] Danecek P. Bonfield J. K. Liddle J. Marshall J. Ohan V. Pollard M. O. Li H. (2021). Twelve years of SAMtools and BCFtools. Gigascience 10:giab008. 10.1093/gigascience/giab008 33590861 PMC7931819

[B18] Danen-Louwerse H. J. Lijklema L. Coenraats M. (1995). Coprecipitation of phosphate with calcium carbonate in Lake Veluwe. Water Res. 29, 1781–1785. 10.1016/0043-1354(94)00301-M

[B19] Doemel W. N. Brock T. D. (1974). Bacterial stromatolites: origin of laminations. Science . 184, 1083–1085. 10.1126/science.184.4141.1083 17736194

[B20] Dupraz C. Reid R. P. Braissant O. Decho A. W. Norman R. S. Visscher P. T. (2009). Processes of carbonate precipitation in modern microbial mats. Earth-Sci. Rev. 96, 141–162. 10.1016/j.earscirev.2008.10.005

[B21] Fazi S. Amalfitano S. Pizzetti I. Pernthaler J. (2007). Efficiency of fluorescence in situ hybridization for bacterial cell identification in temporary river sediments with contrasting water content. Syst. Appl. Microbiol. 30, 463–470. 10.1016/j.syapm.2007.03.003 17452089

[B22] Fazi S. Amalfitano S. Venturi S. Pacini N. Vazquez E. Olaka L. A. Butturini A. (2021). High concentrations of dissolved biogenic methane associated with cyanobacterial blooms in East African lake surface water. Commun. Biol. 4, 845. 10.1038/s42003-021-02365-x 34234272 PMC8263762

[B23] Fernández-Palacios E. Jambrina-Enríquez M. Mentzer S. M. Rodríguez de Vera C. Dinckal A. Égüez N. Mallol C. (2023). Reconstructing formation processes at the Canary Islands indigenous site of Belmaco Cave (La Palma, Spain) through a multiproxy geoarchaeological approach. Geoarchaeology 38, 713–739. 10.1002/gea.21972

[B24] Görgen S. Benzerara K. Skouri-Panet F. Gugger M. Chauvat F. Cassier-Chauvat C. (2021). The diversity of molecular mechanisms of carbonate biomineralization by bacteria. Discover Mat. 1, 1–20. 10.1007/s43939-020-00001-9

[B25] Gupta R. S. (2013). Molecular markers for photosynthetic bacteria and insights into the origin and spread of photosynthesis. Adv. Bot. Res. 66, 37–66. 10.1016/B978-0-12-397923-0.00002-3

[B26] Gupta R. S. Patel S. Saini N. Chen S. (2020). Robust demarcation of 17 distinct Bacillus species clades, proposed as novel *Bacillaceae genera*, by phylogenomics and comparative genomic analyses: description of *Robertmurraya kyonggiensis* sp. nov. and proposal for an emended genus Bacillus limiting it only to the members of the Subtilis and Cereus clades of species. Int. J. Syst. Evol. Microbiol. 70, 5753–5798. 10.1099/ijsem.0.004475 33112222

[B27] Hammes F. Boon N. de Villiers J. Verstraete W. Siciliano S. D. (2003). Strain-specific ureolytic microbial calcium carbonate precipitation. Appl. Environ. Microbiol. 69, 4901–4909. 10.1128/AEM.69.8.4901-4909.2003 12902285 PMC169139

[B28] Han J. Lian B. Ling H. (2013). Induction of calcium carbonate by *Bacillus cereus*. Geomicrobiol. J. 30, 682–689. 10.1080/01490451.2012.75819436854956

[B29] Hsieh T. C. Ma K. H. Chao A. McInerny G. (2016). iNEXT: an R package for rarefaction and extrapolation of species diversity (Hill numbers). Methods Ecol. Evol. 7: 1451–1456. 10.1111/2041-210X.12613

[B30] Imhoff J. F. Rahn T. Künzel S. Neulinger S. C. (2019). Phylogeny of anoxygenic photosynthesis based on sequences of photosynthetic reaction center proteins and a key enzyme in bacteriochlorophyll biosynthesis, the chlorophyllide reductase. Microorganisms 7:576. 10.3390/microorganisms7110576 31752268 PMC6920907

[B31] IPCC (2022). “Climate change 2022: mitigation of climate change,” in Contribution of Working Group III to the Sixth Assessment Report of the Intergovernmental Panel on Climate Change, eds. P. R. Shukla, J. Skea, R. Slade, A. Al Khourdajie, R. van Diemen, D. McCollum, M. Pathak, S. Some, P. Vyas, R. Fradera, M. Belkacemi, A. Hasija, G. Lisboa, S. Luz, and J. Malley (Cambridge; New York, NY: Cambridge University Press). 10.1017/9781009157926

[B32] Kim H. J. Eom H. J. Park C. Jung J. Shin B. Kim W. Park W. (2015). Calcium carbonate precipitation by Bacillus and Sporosarcina strains isolated from concrete and analysis of the bacterial community of concrete. J. Microbiol. Biotechnol. 26, 540–548. 10.4014/jmb.1511.11008 26699752

[B33] Kim S. Y. Sang M. K. Weon H. Y. Jeon Y. A. Ryoo J. H. Song J. (2016). Characterization of multifunctional *Bacillus* sp. GH1-13. The Korean J. Pesticide Sci. 20, 189–196. 10.7585/kjps.2016.20.3.189

[B34] Li H. (2018). Minimap2: pairwise alignment for nucleotide sequences. Bioinformatics 34, 3094–3100. 10.1093/bioinformatics/bty191 29750242 PMC6137996

[B35] Liu G. H. Narsing Rao M. P. Dong Z. Y. Wang J. P. Chen Z. Liu B. Li W. J. (2019). Two novel alkaliphiles, *Bacillus alkalisoli* sp. nov., and *Bacillus solitudinis sp. nov.*, isolated from saline-alkali soil. Extremophiles 23, 759–764. 10.1007/s00792-019-01127-2 31538256

[B36] López-García P. Kazmierczak J. Benzerara K. Kempe S. Guyot F. Moreira D. (2005). Bacterial diversity and carbonate precipitation in the giant microbialites from the highly alkaline Lake Van, Turkey. Extremophiles 9, 263–274. 10.1007/s00792-005-0457-0 15959626

[B37] Lupini G. Proia L. Di Maio M. Amalfitano S. Fazi S. (2011). CARD–FISH and confocal laser scanner microscopy to assess successional changes of the bacterial community in freshwater biofilms. J. Microbiol. Methods 86, 248–251. 10.1016/j.mimet.2011.05.011 21621565

[B38] Mahnic A. Breznik V. Bombek Ihan M. Rupnik M. (2021). Comparison between cultivation and sequencing based approaches for microbiota analysis in swabs and biopsies of chronic wounds. Front. Med. 8:607255. 10.3389/fmed.2021.607255 34150786 PMC8211761

[B39] Marcy Y. Ouverney C. Bik E. M. Lösekann T. Ivanova N. Martin H. G. Quake S. R. (2007). Dissecting biological “dark matter” with single-cell genetic analysis of rare and uncultivated TM7 microbes from the human mouth. Proc. Nat. Acad. Sci. 104, 11889–11894. 10.1073/pnas.0704662104 17620602 PMC1924555

[B40] Marquis R. E. Shin S. Y. (1994). Mineralization and responses of bacterial spores to heat and oxidative agents. FEMS Microbiol. Rev. 14, 375–379. 10.1111/j.1574-6976.1994.tb00111.x 7917424

[B41] Marvasi M. Gallagher K. L. Martinez L. C. Molina Pagán W. C. Rodríguez Santiago R. E. Castilloveitía Vega G. Visscher P. T. (2012). Importance of B4 medium in determining organomineralization potential of bacterial environmental isolates. Geomicrobiol. J. 29, 916–924. 10.1080/01490451.2011.636145

[B42] Meier H. Amann R. Ludwig W. Schleifer K. H. (1999). Specific oligonucleotide probes for in situ detection of a major group of gram-positive bacteria with low DNA G+ C content. Syst. Appl. Microbiol. 22, 186–196. 10.1016/S0723-2020(99)80065-4 10390869

[B43] Mitchell A. C. Dideriksen K. Spangler L. H. Cunningham A. B. Gerlach R. (2010). Microbially enhanced carbon capture and storage by mineral-trapping and solubility-trapping. Environ. Sci. Technol. 44, 5270–5276. 10.1021/es903270w 20540571

[B44] Moore D. M. Reynolds R. C. Jr. (1997). X-Ray Diffraction and the Identification and Analysis of Clay Minerals, 2nd Edn. Oxford: Oxford University Press.

[B45] Moore L. S. Burne R. V. (1987). Microbialites: organosedimentary deposits of benthic microbial communities. Palaios 2:14674. 10.2307/3514674

[B46] Nair S. Li C. Mou S. Zhang Z. Zhang Y. (2022). A novel phage indirectly regulates diatom growth by infecting a diatom-associated biofilm-forming bacterium. Appl. Environ. Microbiol. 88, e02138–e02121. 10.1128/aem.02138-21 35020448 PMC8904054

[B47] Okyay T. O. Nguyen H. N. Castro S. L. Rodrigues D. F. (2016). CO2 sequestration by ureolytic microbial consortia through microbially-induced calcite precipitation. Sci. Total Environ. 572, 671–680. 10.1016/j.scitotenv.2016.06.199 27524723

[B48] Okyay T. O. Rodrigues D. F. (2015). Biotic and abiotic effects on CO2 sequestration during microbially-induced calcium carbonate precipitation. FEMS Microbiol. Ecol. 91:fiv017. 10.1093/femsec/fiv017 25764465

[B49] Pace A. Bourillot R. Bouton A. Vennin E. Galaup S. Bundeleva I. Visscher P. T. (2016). Microbial and diagenetic steps leading to the mineralisation of Great Salt Lake microbialites. Sci. Rep. 6:31495. 10.1038/srep31495 27527125 PMC4985759

[B50] Parada A. E. Needham D. M. Fuhrman J. A. (2016). Every base matters: assessing small subunit rRNA primers for marine microbiomes with mock communities, time series and global field samples. Environ. Microbiol. 18, 1403–1414. 10.1111/1462-2920.13023 26271760

[B51] Park J. Park B. S. Wang P. Patidar S. K. Kim J. H. Kim S. H. Han M. S. (2017). Phycospheric native bacteria *Pelagibaca bermudensis* and *Stapp*ia sp. ameliorate biomass productivity of *Tetraselmis striata* (KCTC1432BP) in co-cultivation system through mutualistic interaction. Front. Plant Sci. 8:289. 10.3389/fpls.2017.00289 28321229 PMC5337489

[B52] Patel S. Gupta R. S. (2020). A phylogenomic and comparative genomic framework for resolving the polyphyly of the genus Bacillus: Proposal for six new genera of Bacillus species, *Peribacillus gen*. nov., *Cytobacillus gen*. nov., *Mesobacillus gen*. nov., *Neobacillus gen*. nov., *Metabacillus gen*. nov. and *Alkalihalobacillus gen*. nov. Int. J. Syst. Evol. Microbiol. 70, 406–438. 10.1099/ijsem.0.003775 31617837

[B53] Pierson B.K. Castenholz R.W. (1992). “The family chloroflexaceae,” in The Prokaryotes, eds A. Balows, H. G. Trüper, M. Dworkin, W. Harder, K. H. Schleifer (New York, NY: Springer).

[B54] Ramm J. Lupu A. Hadas O. Ballot A. Rücker J. Wiedner C. Sukenik A. (2012). A CARD-FISH protocol for the identification and enumeration of cyanobacterial akinetes in lake sediments. FEMS Microbiol. Ecol. 82, 23–36. 10.1111/j.1574-6941.2012.01401.x 22537189

[B55] Raniro H. R. Teles A. P. B. Adam C. Pavinato P. S. (2022). Phosphorus solubility and dynamics in a tropical soil under sources derived from wastewater and sewage sludge. J. Environ. Manage. 302:113984. 10.1016/j.jenvman.2021.113984 34700086

[B56] Reid R. P. Suosaari E. P. Oehlert A. M. Pollier C. G. Dupraz C. (2024). Microbialite accretion and growth: lessons from Shark Bay and the Bahamas. Ann. Rev. Mar. Sci. 16, 487–511. 10.1146/annurev-marine-021423-124637 38231736

[B57] Rhoads D. D. Cox S. B. Rees E. J. Sun Y. Wolcott R. D. (2012). Clinical identification of bacteria in human chronic wound infections: culturing vs. 16S ribosomal DNA sequencing. BMC Infect. Dis. 12:321. 10.1186/1471-2334-12-321 23176603 PMC3542000

[B58] Riding R. (2000). Microbial carbonates: the geological record of calcified bacterial–algal mats and biofilms. Sedimentology 47, 179–214. 10.1046/j.1365-3091.2000.00003.x

[B59] Riding R. (2006). Microbial carbonate abundance compared with fluctuations in metazoan diversity over geological time. Sediment. Geol. 185, 229–238. 10.1016/j.sedgeo.2005.12.015

[B60] Robeson M. S. O'Rourke D. R. Kaehler B. D. Ziemski M. Dillon M. R. Foster J. T. Bokulich N. A. (2021). RESCRIPt: reproducible sequence taxonomy reference database management. PLoS Comput. Biol. 17:e1009581. 10.1371/journal.pcbi.1009581 34748542 PMC8601625

[B61] Rodriguez-Navarro C. Rodriguez-Gallego M. Ben Chekroun K. Gonzalez-Muñoz M. T. (2003). Conservation of ornamental stone by *Myxococcus xanthus*-induced carbonate biomineralization. Appl. Environ. Microbiol. 69, 2182–2193. 10.1128/AEM.69.4.2182-2193.2003 12676699 PMC154787

[B62] Saghaï A. Zivanovic Y. Zeyen N. Moreira D. Benzerara K. Deschamps P. López-García P. (2015). Metagenome-based diversity analyses suggest a significant contribution of non-cyanobacterial lineages to carbonate precipitation in modern microbialites. Front. Microbiol. 6:797. 10.3389/fmicb.2015.00797 26300865 PMC4525015

[B63] Silveira C. B. Thompson F. (2014). 4 the family Alcanivoraceae. Eugene Rosenberg, 459:9783642389221. 10.1007/978-3-642-38922-1_369

[B64] Sorokin D. Y. Chernyh N. A. (2017). *Desulfonatronospira sulfatiphila* sp. nov., and *Desulfitispora elongata* sp. nov., two novel haloalkaliphilic sulfidogenic bacteria from soda lakes. Int. J. Syst. Evol. Microbiol. 67, 396–401. 10.1099/ijsem.0.001640 27902279

[B65] Souza-Egipsy V. Wierzchos J. Ascaso C. Nealson K. H. (2005). Mg–silica precipitation in fossilization mechanisms of sand tufa endolithic microbial community, Mono Lake (California). Chem. Geol. 217, 77–87. 10.1016/j.chemgeo.2004.12.004

[B66] Sun B. Zhao H. Zhao Y. Tucker M. E. Han Z. Yan H. (2020). Bio-precipitation of carbonate and phosphate minerals induced by the bacterium *Citrobacter freundii* ZW123 in an anaerobic environment. Minerals 10:65. 10.3390/min10010065

[B67] Thiel V. Tank M. Bryant D. A. (2018). Diversity of chlorophototrophic bacteria revealed in the omics era. Annu. Rev. Plant Biol. 69, 21–49. 10.1146/annurev-arplant-042817-040500 29505738

[B68] Tytgat B. Verleyen E. Obbels D. Peeters K. De Wever A. D'hondt S. Willems A. (2014). Bacterial diversity assessment in Antarctic terrestrial and aquatic microbial mats: a comparison between bidirectional pyrosequencing and cultivation. PLoS ONE 9:e97564. 10.1371/journal.pone.0097564 24887330 PMC4041716

[B69] Ventura M. Callegari M. L. Morelli L. (2000). S-layer gene as a molecular marker for identification of *Lactobacillus helveticus*. FEMS Microbiol. Lett. 189, 275–279. 10.1111/j.1574-6968.2000.tb09243.x 10930751

[B70] Venturi S. Crognale S. Di Benedetto F. Montegrossi G. Casentini B. Amalfitano S. Fazi S. (2022). Interplay between abiotic and microbial biofilm-mediated processes for travertine formation: insights from a thermal spring (Piscine Carletti, Viterbo, Italy). Geobiology 20, 837–856. 10.1111/gbi.12516 35942584

[B71] Vuong T. T. Kwon B. R. Eom J. I. Shin B. K. Kim S. M. (2020). Interaction between marine bacterium *Stappia* sp. K01 and diatom *Phaeodactylum tricornutum* through extracellular fatty acids. J. Appl. Phycol. 32, 71–82. 10.1007/s10811-019-01931-5

[B72] Warden J. G. Coshell L. Rosen M. R. Breecker D. O. Ruthrof K. X. Omelon C. R. (2019). The importance of groundwater flow to the formation of modern thrombolitic microbialites. Geobiology 17, 536–550. 10.1111/gbi.12344 31119865

[B73] Warnecke F. Luginbühl P. Ivanova N. Ghassemian M. Richardson T. H. Stege J. T. Leadbetter J. R. (2007). Metagenomic and functional analysis of hindgut microbiota of a wood-feeding higher termite. Nature 450, 560–565. 10.1038/nature06269 18033299

[B74] Watson C. Clemens J. Wichern F. (2020). Hazenite: a new secondary phosphorus, potassium and magnesium fertiliser. Plant Soil Environ. 66, 1–14. 10.17221/492/2019-PSE

[B75] Webb G. E. Kamber B. S. (2011). Trace element geochemistry as a tool for interpreting microbialites. Earliest Life Earth Hab. Environ. Methods Detec. 22, 127–170. 10.1007/978-90-481-8794-2_6

[B76] Weber C. F. King G. M. (2007). Physiological, ecological, and phylogenetic characterization of Stappia, a marine CO-oxidizing bacterial genus. Appl. Environ. Microbiol. 73, 1266–1276. 10.1128/AEM.01724-06 17142374 PMC1828652

[B77] Yang H. Martinelli L. Tasso F. Sprocati A. R. Pinzari F. Liu Z. Sun H. J. (2014). A new biogenic, struvite-related phosphate, the ammonium-analog of hazenite, (NH_4_) NaMg_2_ (PO4) 2· 14H_2_O. Am. Mineral. 99, 1761–1765. 10.2138/am.2014.4768

[B78] Yang H. Sun H. J. Downs R. T. (2011). Hazenite, KNaMg_2_ (PO_4_) 2· 14H_2_O, a new biologically related phosphate mineral, from Mono Lake, California, USA. Am. Mineral. 96, 675–681. 10.2138/am.2011.3668

[B79] Yilmaz P. Parfrey L. W. Yarza P. Gerken J. Pruesse E. Quast C. Glöckner F. O. (2014). The SILVA and “all-species living tree project (LTP)” taxonomic frameworks. Nucleic Acids Res. 42, D643–D648. 10.1093/nar/gkt1209 24293649 PMC3965112

[B80] Zeyen N. Benzerara K. Beyssac O. Daval D. Muller E. Thomazo C. Duprat E. (2021). Integrative analysis of the mineralogical and chemical composition of modern microbialites from ten Mexican lakes: What do we learn about their formation?. Geochim. Cosmochim. Acta 305, 148–184. 10.1016/j.gca.2021.04.030

[B81] Zeyen N. Benzerara K. Li J. Groleau A. Balan E. Robert J. L. López-García P. (2015). Formation of low-T hydrated silicates in modern microbialites from Mexico and implications for microbial fossilization. Front. Earth Sci. 3:64. 10.3389/feart.2015.00064

[B82] Zhang Y. Guo Y. J. Dai J. Y. Zhao L. Wu L. P. (2022). Fabrication of hazenite conversion coating on AZ31 Mg alloy. Surface Coatings Technol. 435:128249. 10.1016/j.surfcoat.2022.128249

[B83] Zinder S. H. Mah R. A. (1979). Isolation and characterization of a thermophilic strain of Methanosarcina unable to use H_2_-CO_2_ for methanogenesis. Appl. Environ. Microbiol. 38, 996–1008. 10.1128/aem.38.5.996-1008.1979 16345468 PMC243620

