# Supplementary material for: Carbonate precipitation and phosphate trapping by microbialite isolates from an alkaline insular lake (Bagno dell'Acqua, Pantelleria Island, Italy)
Source: Front Microbiol. 2024 May 22;15:1391968. doi: 10.3389/fmicb.2024.1391968 (PMC11150794; doi:10.3389/fmicb.2024.1391968)
Supplement: Supplementary file 1 [file Table_1.DOCX]

Supplementary Material

Carbonate precipitation and phosphate trapping by microbialite isolates from an alkaline insular lake (Bagno dell’Acqua, Pantelleria Island, Italy).

Cristina Mazzoni*, Agnese Piacentini, Letizia Di Bella, Luca Aldega, Cristina Perinelli, Aida Maria Conte, Michela Ingrassia, Tania Ruspandini, Andrea Bonfanti, Benedetta Caraba, Francesco Giuseppe Falese, Francesco Latino Chiocci, Stefano Fazi*

*** Correspondence:**Corresponding Author
cristina.mazzoni@uniroma1.it; stefano.fazi@irsa.cnr.it

## Supplementary Figures


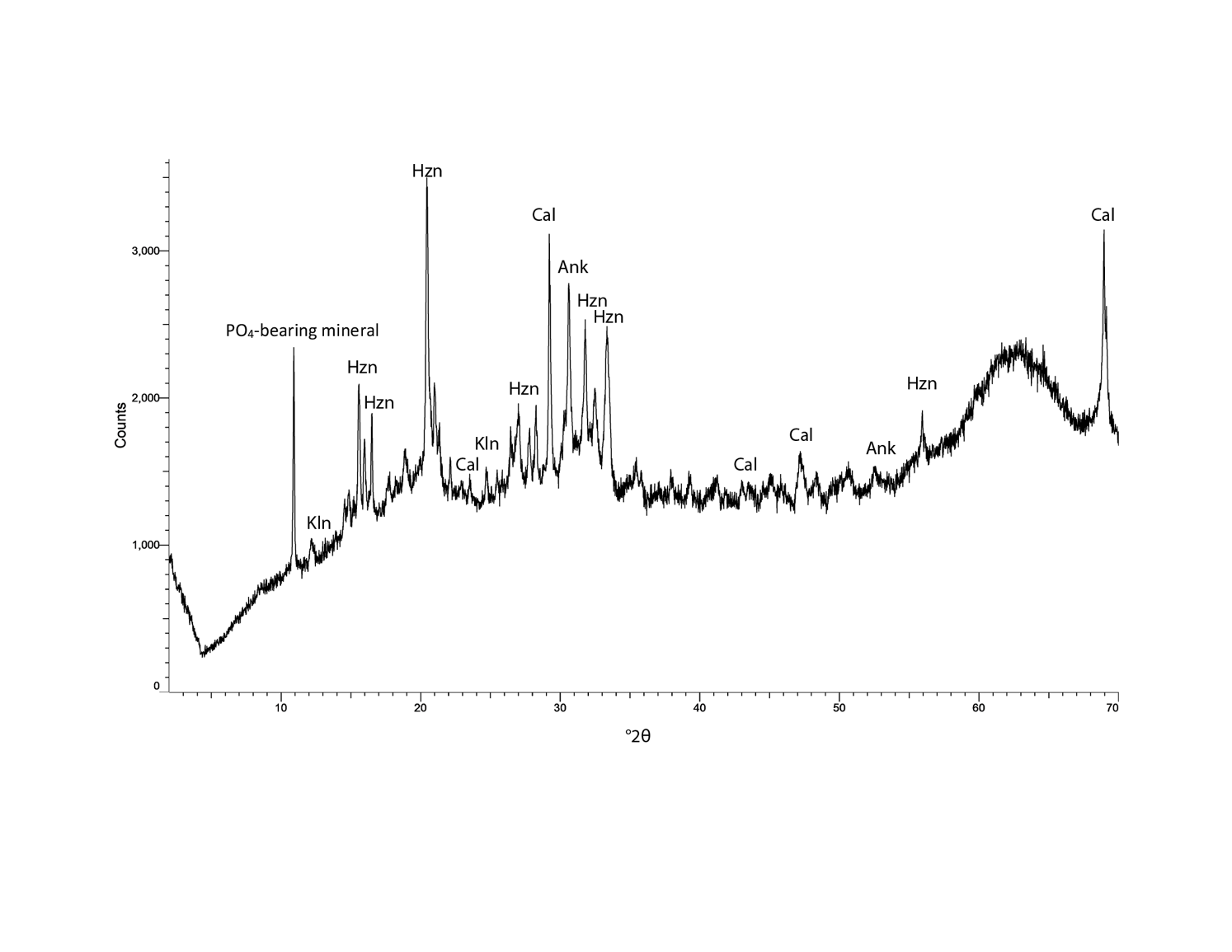


**Supplementary Figure 1.** Selected X-ray diffraction pattern for the crystals precipitated from Stappia sp. 3bis2 strain after 35 days inn B4LW media (Table 4 and figure 6A). Cal-calcite, Ank-ankerite, Hzn-Hazenite, Kln-Kaolinite.

## Supplementary Figure2


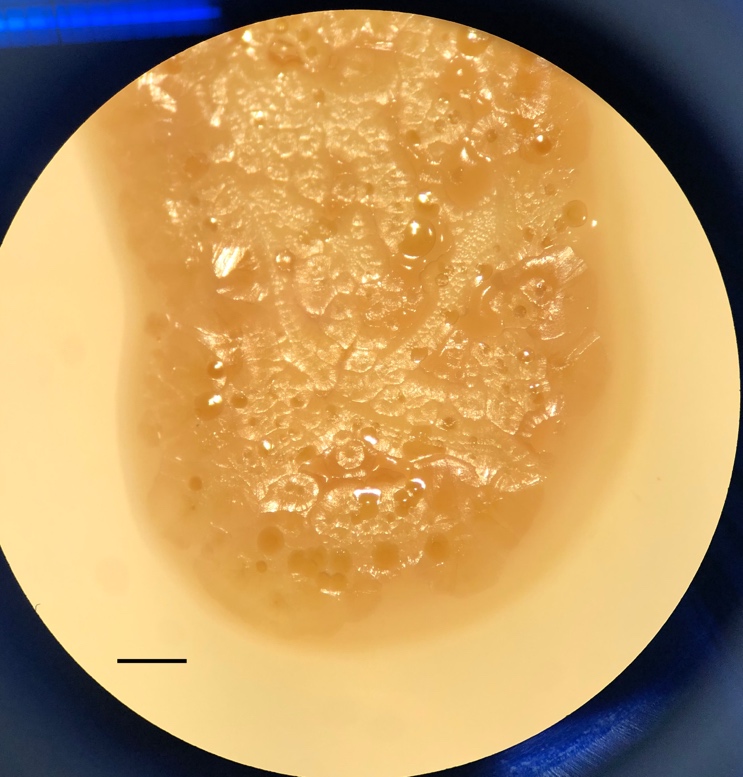


**Supplementary Figure 2.** Crystals are associated within bacterial culture. Cells from saturated culture of *Stappia sp*. 3bis2 strain were spotted onto B4CaCl_2_ pH8, incubated at 28°C for 11 days and observed under stereomicroscope. Scale bar: 1mm

## Supplementary Figure3


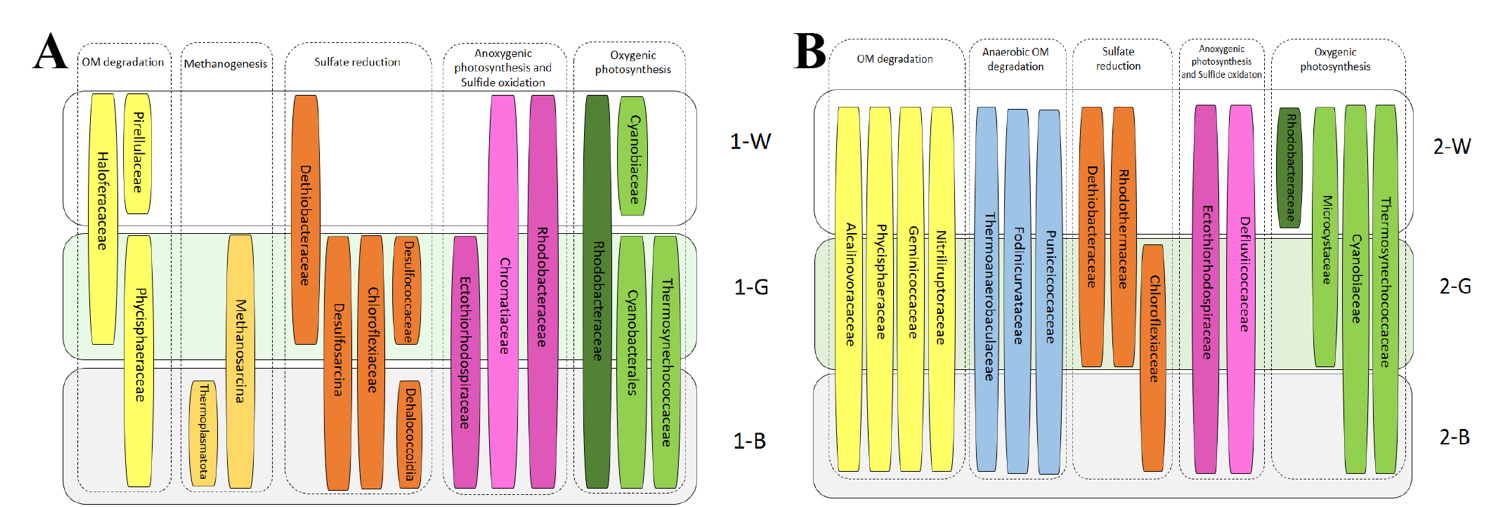


**Supplementary Figure 3.** Diagram summarizing the functionality of the most abundant families belonging to phyla with a relative abundance of reads greater than 5%. (A) Emerged microbialite with the main families present in the layers (surface 1-W; intermediate 1-G; deep 1-B); (B) Submerged microbialite with the main families present in the three different layers (surface 2-W; intermediate 2-G; deep 2-B).
